# Role of the Character of the Excited State of Singly Reduced Rh_2_(II,II) Intermediates on Photocatalytic Activity

**DOI:** 10.1021/jacs.6c02339

**Published:** 2026-04-15

**Authors:** Piyush Gupta, Curtis E. Moore, Claudia Turro

**Affiliations:** Department of Chemistry and Biochemistry, 2647 The Ohio State University, Columbus, Ohio 43214, United States

## Abstract

Singly reduced intermediates have recently been implicated as photoactive intermediates in a number of important reactions; however, their photophysical properties remain poorly understood. A series of dirhodium­(II,II) complexes, *cis-*[Rh_2_(*p-*R-Form)_2_(bncn)_2_]^2+^ (bncn = benzo­[*c*]­cinnoline; *p-*R-Form = *N,N*′*-*di-*p*-R-phenylformamidinate), where R = −OCH_3_ (**1**), −CH_3_ (**2**), −H (**3**), −F (**4**), –Cl (**5**), and −CF_3_ (**6**), and their respective radical anions were prepared and their excited state properties were investigated. Complex **3** acted as a single-molecule photocatalyst for H_2_ production with red light. Substitution on the formamidinate ligands in **1**–**6** affected the energy of the Rh_2_(δ*)/Form­(π,nb) highest occupied molecular orbital (HOMO), consistent with the metal/ligand-to-ligand charge transfer (^1^ML-LCT) absorption maxima and the ^3^ML-LCT excited state lifetime, ranging from 1.6 ns in **1** to 54 ns in **6** in CH_3_CN. The highest turnover number for photocatalytic H_2_ evolution was observed for **3**, and the lowest values were for **1** and **6**. The radical anion, [Rh_2_]^−^, formed during photocatalysis, was shown to absorb a photon and undergo a second reduction. The lifetimes of the doublet excited states of [**3**]^−^ and [**6**]^−^ were 0.49 and 0.24 ns, respectively. Calculations showed that the lowest energy excited state in [**3**]^−^ was ^2^ML-LCT, whereas that in [**6**]^−^ was a bncn^–^ → Rh_2_(σ*) ligand-to-metal charge transfer (^2^LMCT) state. The ^2^LMCT state stabilized across the series from [**1**]^−^ to [**6**]^−^, pointing at its role in modulating the photophysical properties. This work highlights the importance of the reductive quenching of [Rh_2_]^−^ and the generation of the doubly reduced species to effectively catalyze hydrogen evolution.

## Graphical abstract

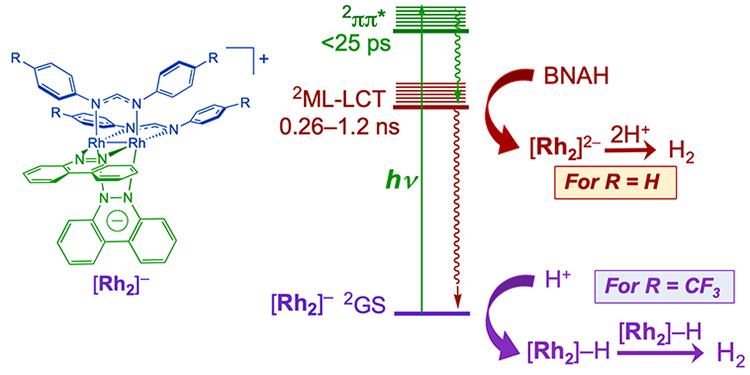

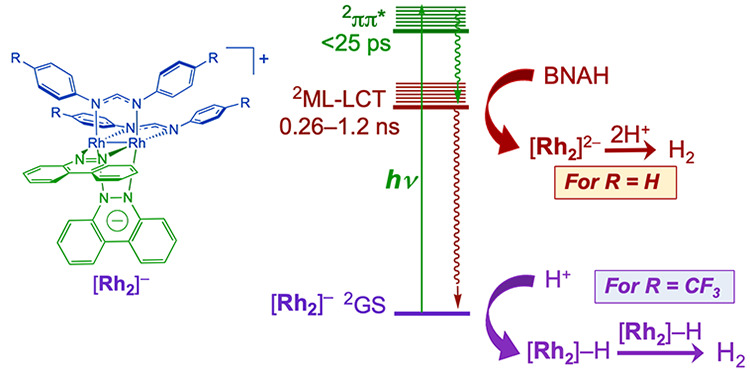

## Introduction

Photochemically driven catalytic reactions that result in the formation of new chemical bonds have been intensely investigated in recent years because they are able to use the energy of photons to generate fuels or to create value-added chemicals.^1–10^ Recently, the Turro group reported a new class of air-stable and robust single-molecule dirhodium­(II,II) photocatalysts, including *cis-*[Rh_2_(DPhF)_2_(bncn)_2_]^2+^ (**3**; DPhF = *N,N*′-diphenylformamidinate, bncn = benzo­[*c*]­cinnoline; Figure 1, R = H). These complexes produce H_2_ upon irradiation with low-energy red/near-IR irradiation and feature turnover numbers, TONs, of ∼200 (24 h, λ_irr_ = 670 nm).^11,12^ A salient mechanism of these Rh_2_(II,II) photocatalysts, [Rh_2_], is that they undergo the sequential absorption of two photons, such that the corresponding singly reduced complexes, [Rh_2_]^−^, are also photoactive.

**Figure 1:**
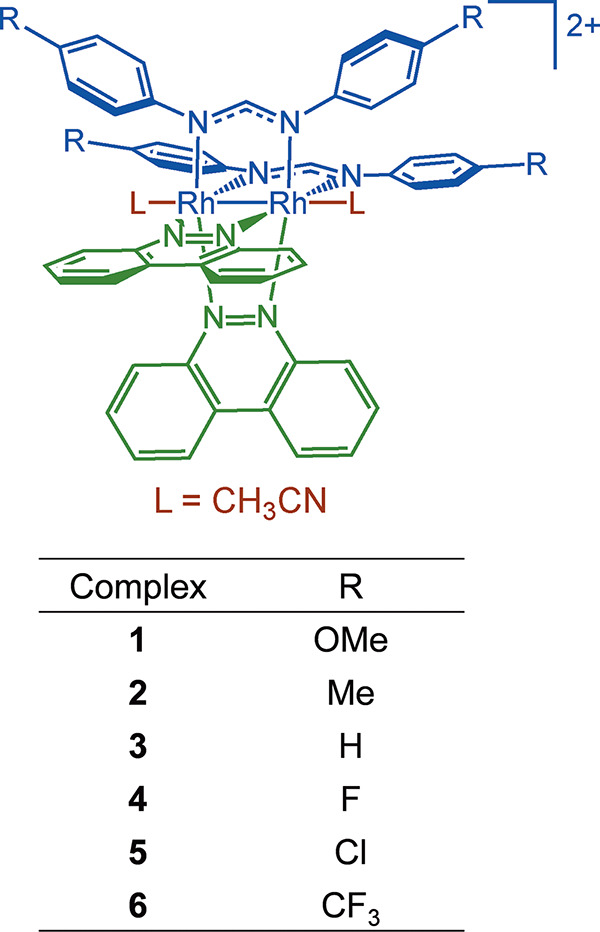
Schematic representation of the molecular structures of **1**–**6**.

Since the publication of the mechanism of the dirhodium­(II,II) photocatalysts involving the excited state reactivity of the singly reduced complex,^11a^ a number of reports have appeared in the literature where a reduced transition metal intermediate has been invoked in photoredox catalysis.^13–15^ The excited states of these intermediates are expected to be strongly reducing, although in the case of organic radical anions, their doublet excited states have been shown to be short-lived.^16^ In addition, it was found that, in some cases, the reactive species was not the organic radical anion, but was later identified as a decomposition product, the corresponding closed-shell doubly reduced species, or solvated electrons.^16,17^

In contrast to organic anions and radicals, there are only a handful of reduced inorganic complexes shown to participate in photocatalysis, consisting of Ir­(III) and Ru­(II) complexes. In one case, the long-lived triplet metal/ligand-to-ligand charge transfer (^3^ML-LCT) excited state of [Ir­(ppy)_2_(bpy)]^+^ (ppy = 2-phenylpridinato, bpy = 2,2′-bipyridine) is reductively quenched to form a charge transfer complex containing the one-electron reduced [Ir­(ppy)_2_(bpy^•–^)].^14^ The latter is able to transfer an electron to aryl halides upon excitation with visible light, resulting in hydrodehalogenation.^14^ The excited state properties of the related series [Ir­(dFCF_3_ppy)_2_(R_2_-bpy)]^+^ (dFCF_3_ppy = 2-(2,4-difluorophenyl)-4-trifluoromethylpyridine), where R_2_-bpy represents substituted bpy ligands with *R*_2_– = 4,4′-(OCH_3_)_2_–, 4,4′-^t^Bu–, 4,4′-CO_2_CH_3_–, 4,4′-(CF_3_)_2_–, and 5,5′-(CF_3_)_2_–, were investigated following their chemical reduction to generate the corresponding neutral complex with the electron on the respective R_2_-bpy ligand.^13^ The doublet excited state lifetimes of the [Ir­(dFCF_3_ppy)_2_(R_2_-bpy^•–^)] series range from 16 to 22 ps, such that the observed bimolecular reactivity is believed to arise from preassociation of the complex and the substrate.^13^ The reduction of [Ru­(bpy)_3_]^2+^ generates [Ru­(bpy)_2_(bpy^•–^)]^+^, which is able to transfer an electron to aryl halides upon steady-state irradiation, resulting in the corresponding aryl radical.^15^

In Rh_2_(II,II) photocatalysts, the photoactivation of [Rh_2_]^−^ is a critical step in the mechanism. Recent work has shown that the reductive quenching of the ^3^ML-LCT state of [Rh_2_] complexes by BNAH (1-benzyl-1,4-dihydronicotamide) to produce [Rh_2_]^−^ is the first step in the photocatalytic cycle.^11,12^ Electrochemical results and theoretical calculations using **3** have shown that the bncn ligand serves as the active site for reduction and protonation, leading to H_2_ evolution, without the formation of a rhodium-hydride intermediate.^12^ However, the short lifetimes of the *­[Rh_2_]^−^ intermediates represent a serious drawback that limits the TONs and turnover frequencies (TOFs) of this class of photocatalysts. Therefore, a better understanding of the molecular structural and electronic properties required to extend the excited state lifetimes of *­[Rh_2_]^−^ is needed. To this end, the present work focuses on a series of dirhodium­(II,II) complexes, *cis-*[Rh_2_(*p-*R-Form)_2_(bncn)_2_]^2+^ (*p-*R-Form = *N,N*′*-*di-*para*-R-phenylformamidinate), where R = −OCH_3_ (**1**), −CH_3_ (**2**), −H (**3**), −F (**4**), −Cl (**5**), and −CF_3_ (**6**), to investigate their excited state properties and those of the singly reduced species, [**1**]^−^–[**6**]^−^. The molecular structures of **1**–**6** are shown in Figure 1. Within this series, longer excited state lifetimes of the [Rh_2_] complexes did not result in higher catalytic activity, prompting the examination of the photophysical properties of the corresponding [Rh_2_]^−^ complexes. The latter show that the nature of the excited state plays a significant role in the lifetime and subsequent reactivity. These results highlight the role of the excited states of the singly reduced intermediates in the photocatalytic cycle for H_2_ production, providing important information for the design of efficient transition metal photocatalysts.

## Experimental Section

### Materials

All materials were used as received unless otherwise noted. Rh_2_(μ*–*OAc)_4_ was purchased from Pressure Chemicals and *cis*–[Rh_2_(DPhF)_2_(bncn)_2_]^2+^ (**3**; DPhF = *N,N*′-diphenylformamidinate) was synthesized using the previously reported method.^12b^ *N,N*′-di-*p*-R-arylformamides (*p*-R-HForm, where R = −OCH_3_, –CH_3_, −F, −Cl, −CF_3_) were prepared using a literature method, by refluxing the respective aniline and triethyl orthoformate in the presence of a catalytic amount of glacial acetic acid for 3 h.^18,19^ *N,N*′-Diphenylformamide (HDPhF) was purchased from Sigma-Aldrich and recrystallized in hexanes before use. Benzo­[*c*]­cinnoline (bncn) was obtained from Wako Chemicals. The aniline analogs, bis­(cyclopentadienyl)­cobalt­(II), triethyl orthoformate, Et_3_OBF_4_ (1 M in dichloromethane), 1,2-dichloroethane, electrolytic grade tetrabutylammonium hexafluorophosphate (TBAPF_6_, >99.0%), and spectroscopic grade acetonitrile were obtained from Sigma-Aldrich. The solvents hexanes, acetone, and diethyl ether were obtained from Fisher Scientific. Acetonitrile was dried over calcium hydride, while *N,N*′-dimethylformamide (DMF) and dichloromethane were purified using a MBRAUN MB-SPS solvent purification system. *cis*-[Rh_2_(*p-*R-Form)_2_(bncn)_2_]^2+^ (R = −OCH_3_, −CH_3_, −F, −Cl, −CF_3_) were synthesized following the three-step method described for **3**, starting with the corresponding formamidinate ligand, as illustrated in Scheme 1 with the synthetic details and ^1^H NMR data in the Supporting Information.

**Scheme 1:**
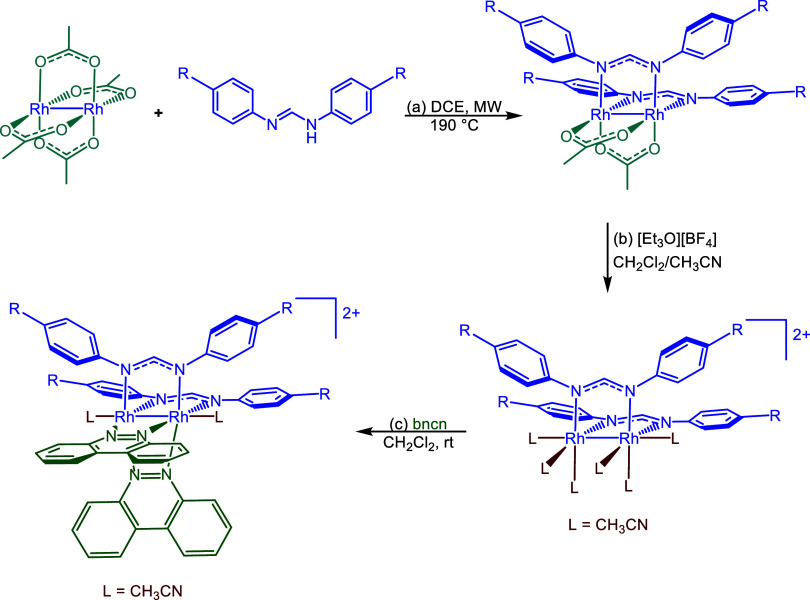
General Synthetic Scheme for *cis-*[Rh_2_(*p*-R-Form)_2_(bncn)_2_]­[BF_4_]_2_ Complexes **1**–**6**

### Instrumentation and Methods

Microwave synthesis was carried out in a CEM Discover microwave reactor in 10 mL sealed CEM sample tubes. ^1^H NMR spectra were obtained using a Bruker Avance NEO 400 spectrometer. Steady-state emission spectra were recorded on a Fluorolog-3 (Horiba) spectrometer at 77 K in acetonitrile using a setup described previously.^12b^ An EC Epsilon EClipse potentiostat/galvanostat/bipotentiostat (BASi) was used for electrochemical measurements with 100 mM TBAPF_6_ as the supporting electrolyte in CH_3_CN and with a 3 mm glassy carbon disk working electrode, a Pt wire counter electrode, and a Ag/AgCl (in 3 M NaCl) reference electrode. Photocatalysis studies were carried out in 3 mL of DMF in a 1 cm × 1 cm airtight quartz cell using the setup previously described,^12b^ containing 100 mM *p*-tolylsulfonic acid (TsOH), 30 mM 1-benzyl-1,4-dihydronicotamide (BNAH), and the dirhodium photocatalyst. The concentration of the dirhodium complex was adjusted to attain an absorbance of ∼0.5 at the irradiation wavelength. Four 595 nm light-emitting diodes (LEDs), purchased from Luxeon Star LEDs, were used at their maximum power of 50 mW as the irradiation source. Hydrogen evolution was measured using gas chromatography on a Shimadzu GC-2014 with argon as the carrier gas.

Excited state dynamics were investigated using nanosecond and femtosecond transient absorption spectroscopy, nsTA and fsTA, respectively. The samples were prepared in acetonitrile with an absorbance of ∼0.4 at the excitation wavelength, λ_exc_, and electronic absorption spectra were recorded before and after each TA experiment to ensure no decomposition occurred from the high-energy laser pulses. The fsTA experiments were conducted using a previously reported Ti:sapphire laser system and amplifier (Astrella, Coherent).^20^ The pump laser wavelength was generated using an optical parametric amplifier (OPerA Solo, Coherent), resulting in powers of the laser beam at the sample of 4–8 mW at 600 nm and ∼12 mW at 650 nm. TA samples for **1**–**6** were prepared using a 1 mm × 1 cm quartz cuvette under an ambient atmosphere. The singly reduced complexes were prepared under a N_2_ atmosphere in a 1 mm × 1 cm quartz cuvette fused with a Kontes top by reducing the respective dicationic complexes with one equivalent of cobaltocene. An Edinburgh LP980 Spectrometer equipped with a PMT detector for single wavelength measurements and iCCD-based broad band capability was used for nsTA experiments, where the tunable BasiScan OPO (Spectra Physics) pumped by a Nd:YAG laser (Spectra Physics, INDI-40) was used to excite the samples, as previously described.^11^ The samples for nsTA experiments were prepared in 1 cm × 1 cm quartz cuvettes fused with a Kontes-top valve and purged with N_2_ before each measurement.

Density functional theory (DFT) calculations were undertaken using the Gaussian 16 package with the B3LYP functional.^21,22^ The SDD basis set and effective core potential were used for rhodium, while the other atoms were treated with the 6–31+G­(d,p) basis set.^23^ The ground state geometries of the dicationic [Rh_2_] and singly reduced [Rh_2_]^−^ species were optimized in CH_3_CN using a CPCM model.^24,25^ Time-dependent DFT (TD-DFT) calculations were used to produce the singlet and doublet excited states for [Rh_2_] and [Rh_2_]^−^ complexes, respectively, to evaluate their frontier molecular orbitals and changes in the lowest energy transitions in these complexes.^26,27^ ChemCraft and GaussView 06 were used to visualize the frontier molecular orbitals.

Single crystals for X-ray diffraction (XRD) analysis were obtained by slow diffusion of diethyl ether in a saturated solution of the respective complexes in acetonitrile at room temperature. The XRD studies were carried out on a Bruker Kappa Photon III CPAD diffractometer equipped with a Mo Kα source (λ = 0.71073 Å). Details of each structure and crystallization are presented in the Supporting Information.

## Results and Discussion

### X-ray Diffraction (XRD) and Electrochemistry

The single crystal XRD structures of **1**, **2**, and **4**–**6** are shown in Figure S1. The dirhodium intermetallic distance varies from 2.387 Å in **6** to 2.410 Å in **2** with no apparent trend across the series (Table S1); these values are similar to that previously reported for **3**, 2.4049 Å.^11a^ The observation of minor structural deviations in a series of neutral dirhodium­(II,II) tetrakis *N,N*′-diarylformamidinate complexes was previously reported by Ren and co-workers.^28^ The axial sites of **1**, **2**, **4**, and **5** were symmetrically coordinated by two acetonitrile solvent molecules, similar to **3**. In **6**, one of the axial sites has 100% CH_3_CN occupancy, while the other exhibits partial occupancy by a water molecule (20%). The different axial coordination in **6** is attributed to the reduced electron density of the Rh_2_ core in the presence of the electron-withdrawing substituents.^29^ The ^1^H NMR spectra of **1**–**6** exhibit the characteristic triplet of dirhodium formamidinate complexes in the 6.94 ppm to 7.04 ppm range, corresponding to the methine proton symmetrically coupled to the two ^103^Rh nuclei. A ∼0.4 ppm upfield shift was observed for the protons positioned *ortho* to the substituent in **1** relative to **3**, whereas a downfield shift of similar magnitude was measured for **6**. These shifts are consistent with the electronic shielding and deshielding effects of −OCH_3_ and −CF_3_ groups, respectively.

The cyclic voltammograms (CVs) of **1**–**6** in CH_3_CN (0.1 M TBAPF_6_) are shown in Figure 2, and the corresponding half-wave potentials, *E*_1/2_, are listed in Table 1. Complexes **2**–**6** exhibit four reversible redox events, whereas an additional quasi-reversible oxidation is observed in **1** (Figure S3). The couple at *E*_1/2_([Rh_2_]^3+/2+^) = +1.28 V vs Ag/AgCl for **3** was previously assigned as the one-electron oxidation of the Rh_2_(δ*)/Form­(π,nb) HOMO.^11a^ A significant shift is observed in the *E*_1/2_([Rh_2_]^3+/2+^) values across the series, as expected based on the decreasing electron density on the formamidinate ligand from **1** to **6**, as previously reported in related tetrakis paddlewheel complexes of the type M_2_(Form)_4_ (M = Rh, Ni, Mo, Ru; Form = formamidinate) with substituted formamidinate ligands.^28,30^ A cathodic shift of 150 mV is observed for **1** relative to **3**, with *E*_1/2_([Rh_2_]^3+/2+^) = +1.13 V vs Ag/AgCl, and a smaller, 50 mV shift is recorded for **2**, with *E*_1/2_([Rh_2_]^3+/2+^) = +1.23 V vs Ag/AgCl, similar to the 200 and 70 mV cathodic shifts reported for the corresponding neutral tetrakis counterparts with respect to Rh_2_(DPhF)_4_.^28b^ The ease in oxidation measured for **1** and **2** compared to **3** indicates a destabilization of the HOMO in the former, attributed to a higher electron density on the Rh_2_(δ*)/Form­(π,nb) MO. Conversely, the anodic shifts of the *E*_1/2_[Rh_2_]^3+/2+^ couples in **4**–**6** by 70–220 mV relative to **3** are consistent with the stabilization of the HOMO as the electron density on the dirhodium core is reduced by the electron-withdrawing substituents. The more pronounced shift observed for **6** has been previously reported in the tetrakis Rh_2_(Form)_4_ series, and a similar trend is also recorded with weaker donating ligands than formamidinates, such as acetates.^11,12,31–33^

**Figure 2:**
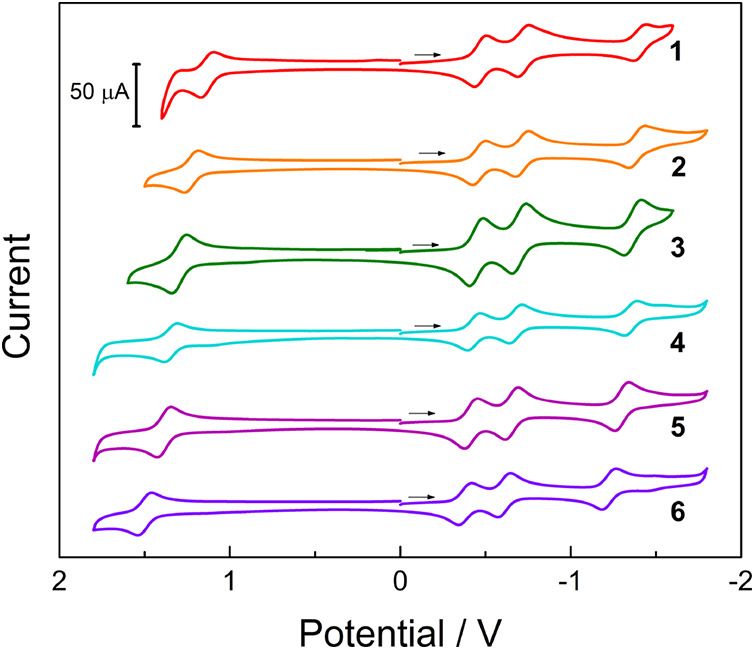
Cyclic voltammetry of **1**–**6** in CH_3_CN (vs Ag/AgCl, 0.1 M TBAPF_6_).

**Table 1:** Hammett Substituent Constants (*σ*_R_), Electronic Absorption Maxima (λ_abs_), Molar Extinction Coefficients, ε, and Electrochemical Half-Wave Reduction Potentials, *E*_1/2_, of **1**–**6** in CH_3_CN

complex	σ_R_ ^a^	λ_abs_/nm (ε × 10^3^ M^–1^ cm^–1^)	*E*_1/2_/V^b^
**1**	–0.27	342(14.4), 353(14.4), 403(8.4), 656(8.6)	+1.13, −0.47, −0.72, −1.40
**2**	–0.17	343(14.6), 404(7.9), 645(8.4)	+1.23, −0.46, −0.71, −1.39
**3**	0	342(13.7), 358(13.3), 403(7.4), 633(8.8)	+1.28, −0.45, −0.70, −1.36
**4**	+0.06	342(13.5), 354(13.4), 404(7.1), 623(9.2)	+1.35, −0.43, −0.68, −1.35
**5**	+0.23	343(13.8), 355(13.8), 403(7.3), 617(9.4)	+1.38, −0.42, −0.65, −1.30
**6**	+0.54	342(13.9), 356(14.3), 401(7.5), 599(10.2)	+1.50, −0.38, −0.61, −1.22

a From ref 34.

b vs Ag/AgCl, 0.1 M TBAPF_6_.

The *E*_1/2_[Rh_2_]^3+/2+^ couples observed for the dicationic **1**–**6** display a significant anodic shift, ∼800 mV, relative to their neutral Rh_2_(Form)_4_ counterparts, which can be attributed to the positive overall charge of the former. Using the Hammett relationship,^34^ Ren et al. and Das et al. quantified the reactivity constant (ρ) for dirhodium­(II,II) tetrakis-formamidinate and -acetate complexes, resulting in ρ = 98 mV and ρ = 84 mV, respectively.^28b,35^ A similar value is calculated for **1**–**6**, ρ = 108 mV, from the plot of *E*_1/2_[Rh_2_]^3+/2+^ in CH_3_CN as a function of the substituent Hammett constant (Figure S2).

A second quasi-reversible oxidation event is observed in **1** at *E*_1/2_([Rh_2_]^4+/3+^) = +1.36 V vs Ag/AgCl (shown in Figure S3) that can be assigned as the removal of an electron from the HOMO–1,^11,12^ also centered on a Rh_2_(δ*)/Form­(π/nb) orbital. This couple has been reported at +0.77 V in Rh_2_(*p*-OMe-Form)_4_, at a potential 210 mV less positive than that of Rh_2_(DPhF)_4_.^28b^ Assuming a similar shift of ∼800 mV for the second oxidation event, the *E*_1/2_[Rh_2_]^4+/3+^ values predicted for **2**–**6** would lie outside the measurement window of the solvent.

Three reversible reduction couples are observed for **3** at −0.45 V, −0.70 V, and −1.36 V vs Ag/AgCl in CH_3_CN (Table 1). The first two events have been previously attributed to the reduction of each of the two bncn ligands, *E*_1/2_(bncn^0/–^), which correspond to the placement of electrons on the LUMO and LUMO+1.^11,12^ Although the LUMO and LUMO + 1 are calculated to be degenerate, it is more difficult to place an electron on the second bncn ligand following the first reduction, shifting that couple to a more negative potential.^11,12^ Similar values were observed in a previously reported complex with an ethoxycarbonyl substitution (σ_R_ = +0.450), *cis*-[Rh_2_(*p*-COOEt-Form)_2_(bncn)_2_]^2+^, where the bncn-centered reduction couples were observed at −0.39 V and −0.59 V vs Ag/AgCl.^31^

The third reduction in **3** has been attributed to the addition of an electron into the Rh_2_(σ*) LUMO+2, which is purely metal-centered.^11,12^ This couple experiences a more pronounced effect as a function of substituent across the series as compared to the bncn-centered reduction events. For example, an anodic shift of 140 mV is observed for **6** relative to **3**, considerably larger than those recorded for the first and second reduction couples in the same complex, 70 mV and 90 mV, respectively. The smaller shift of the bncn-centered reduction potential as a function of substituent, as compared to the larger shift in oxidation associated with the energy of the HOMO, results in an increase of the HOMO–LUMO gap, Δ*E*_exp_, from **1** to **6** (Table S1).

### Electronic Structure Calculations and Electronic Absorption Spectroscopy

The calculated energies of the frontier molecular orbitals for **1**–**6** and the orbital contributions to the HOMO and LUMO of each complex are shown in Figures S4 and S5, respectively. In addition, the HOMO–LUMO energy gaps (Δ*E*_calc_) and the energies of the lowest energy singlet excited states (E_ML‑LCT_^1^) of **1**–**6** are listed in Table S1. The electron density in the HOMO is localized on the Rh_2_(δ*) and a nonbonding π-orbital of the formamidinate ligand, Form­(π,nb), in each complex (Figure S5), as described previously in related systems.^11,12,36^ A 0.53 eV stabilization of the HOMO is calculated from **1** to **6**, in agreement with the anodic shift of the *E*_1/2_[Rh_2_]^3+/2+^ redox couple by 0.37 V in these complexes (Table 1). The HOMO–1 is of similar parentage, Rh_2_(δ*)/Form­(π,nb), at slightly lower energies, ∼0.25 eV, than the respective HOMO of each complex (Figure S4).

The high symmetry of **1**–**6** results in degenerate LUMO and LUMO+1 orbitals that are delocalized over the two bncn ligands (Figure S4), as calculated for related complexes.^11,12^ The calculations show a modest stabilization of 0.13 eV of these orbitals from **1** to **6**, consistent with the experimental anodic shifts of 0.09 and 0.11 V for the first and second reduction couples, respectively, across the series. The magnitude of Δ*E*_calc_ increases by 0.38 eV from **1**–**6**, consistent with the larger HOMO–LUMO gap measured electrochemically, Δ*E*_exp_ (Table S1), 0.28 V. The lowest-energy singlet transitions of **1**–**6** are calculated to arise from the movement of an electron from the HOMO to the LUMO, Rh_2_(δ*)/Form­(π,nb) → bncn­(π*), in agreement with the ^1^ML-LCT absorption reported for Rh_2_(II,II) complexes containing formamidinate and bncn bridging ligands.^11,12^ The energy of the lowest-energy ^1^ML-LCT excited state is calculated to increase from **1** to **6** by 0.34 eV (Table S1), in agreement with the trends in Δ*E*_calc_ and Δ*E*_exp_, as well as the experimentally observed blue shift in the lowest energy absorption maximum across the series described below.

The electronic absorption spectra of **1**–**6** recorded in CH_3_CN are shown in Figure 3, and their corresponding absorption maxima and molar extinction coefficients are listed in Table 1. The absorption features of **3** have been previously assigned, where the lowest energy broad band at 633 nm corresponds to Rh_2_(δ*)/Form­(π*/nb) → bncn­(π*) ^1^ML-LCT, and that at 403 nm is a bncn-centered n → π* transition.^11,12^ The ^1^ML-LCT maximum shifts to higher energies across the series from **1**–**6**, whereas the position of the bncn-centered peak remains relatively unchanged. These findings are in agreement with the electrochemical results, as the bncn-localized reduction couples display a negligible anodic shift in comparison to the *E*_1/2_[Rh_2_]^3+/2+^ values. As the HOMO–LUMO gap increases from **1**–**6** (Table S1), it is evident from Table 1 that the ^1^ML-LCT transition blue-shifts from 656 nm in **1** (ε = 8600 M^–1^ cm^–1^) to 599 nm in **6** (ε = 10,200 M^–1^ cm^–1^).

**Figure 3:**
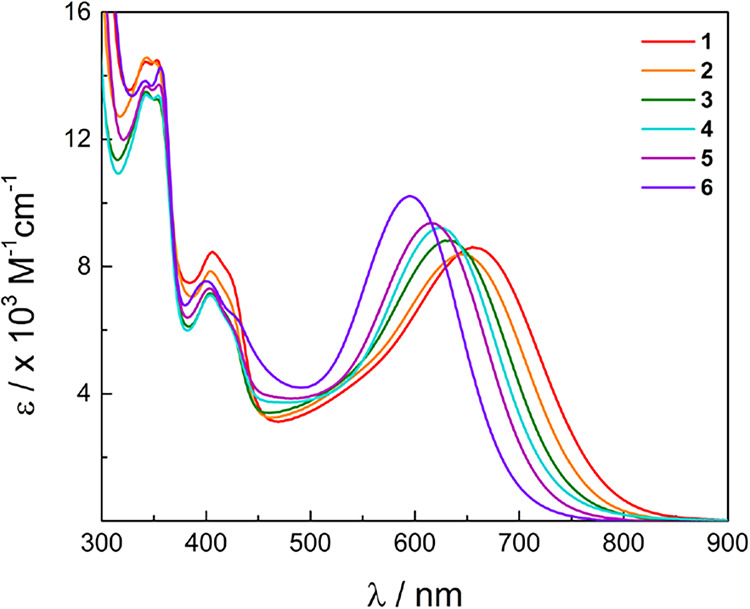
Electronic absorption spectra of **1**–**6** in CH_3_CN.

### Excited State Properties

Luminescence is not commonly observed in dirhodium paddlewheel complexes, but the Turro group reported that **3** and related complexes are emissive at 77 K, assigned to the Rh_2_(δ*)/Form­(π,nb) → bncn­(π*) ^3^ML-LCT state.^11,12^ The 77 K emission and excitation spectra of **1**, **2**, and **4**–**6** in CH_3_CN are shown in Figure S6, and the corresponding maxima, λ_em_, are listed in Table 2. It is evident from Figure S6 that the excitation spectra of each complex overlay with the respective absorption profile, confirming that the observed luminescence arises from the complex and not a highly emissive impurity. A blue shift in the emission maxima is observed from **1**–**6**, similar to that recorded for the ^1^ML-LCT absorption in the series, with λ_em_ = 893 nm for **1**, λ_em_ = 860 nm for **3**, and λ_em_ = 780 nm for **6**. The energy of the ^3^ML-LCT excited states of **1**–**6**, E_00_^T^, derived from the 77 K emission maxima are listed in Table 2 and increase linearly as a function of the Hammett substituent constant, σ_R_, as shown in Figure S7, consistent with the increase in the HOMO–LUMO gap (Table S1) and the ^1^ML-LCT absorption maxima (Table 1).

**Table 2:** Singlet and Triplet Excited State Lifetimes, τ_S_ and τ_T_, Emission Maxima at 77 K, λ_em_, ^3^ML-LCT Excited State Energy, *E*_00_^T^, Excited State Reduction Potential, ^3^E*_red_, and Driving Force for the Reductive Quenching by BNAH, Δ*G*_RQ_, in CH_3_CN and Photocatalytic Turnover Numbers in DMF, TON, for **1**–**6**

complex	τ_S_, τ_T_	λ_em_/nm	E_00_^T^/eV^a^	^3^E*_red_/V	Δ*G*_RQ_/V	TON^d^
**1**	10 ps, 1.6 ns	893	1.40	+0.93	–0.33	12
**2**	10 ps, 9 ns	875	1.42	+0.96	–0.35	91
**3**	15 ps, 19 ns^b^	860^c^	1.44^c^	+0.99	–0.38	125
**4**	6 ps, 17 ns	842	1.47	+1.04	–0.43	88
**5**	9 ps, 22 ns	812	1.53	+1.11	–0.50	62
**6**	11 ps, 54 ns	780	1.59	+1.21	–0.60	12

a vs Ag/AgCl, 0.1 M TBAPF_6_.

b From ref 11b.

c From ref 12b.

d After 24 h of irradiation.

The excited state dynamics of **1**–**6**, [Rh_2_], were investigated using transient absorption spectroscopy with nanosecond and femtosecond time resolution, nsTA and fsTA, respectively. The fsTA spectra recorded for **1** are shown in Figure 4 (λ_exc_ = 400 nm, FWHM = 85 fs), while those of **2** and **4**–**6** are presented in Figure S8, with the corresponding singlet and triplet excited state lifetimes, τ_S_ and τ_T_, of **1**–**6** listed in Table 2. A broad positive signal with a maximum at 445 nm was recorded for **1**, along with an intense ground-state bleach with a minimum at 643 nm (Figure 4). These spectral features are similar to those previously reported for structurally related Rh_2_(II,II) complexes, including **3**, where the excited state absorption was assigned to the reduced bncn ligand in the ML-LCT excited state.^11,12,36a^ For **1**, the decay at 445 nm was fitted with a monoexponential function yielding a lifetime of 1.2(3) ns, while a biexponential fit was required for the bleach recovery, resulting in 10(2) ps and 1.6(6) ns components. The shorter lifetime, τ_S_, has been previously attributed to the ^1^ML-LCT state, which undergoes intersystem crossing to the corresponding triplet, while the longer component, τ_T_, is associated with the decay from the ^3^ML-LCT to the ground state, ^1^GS. The fsTA spectral features and lifetimes measured for **2** and **4**–**6** are similar to those observed for **1** and **3** (Figure S8 and Table 2).

**Figure 4:**
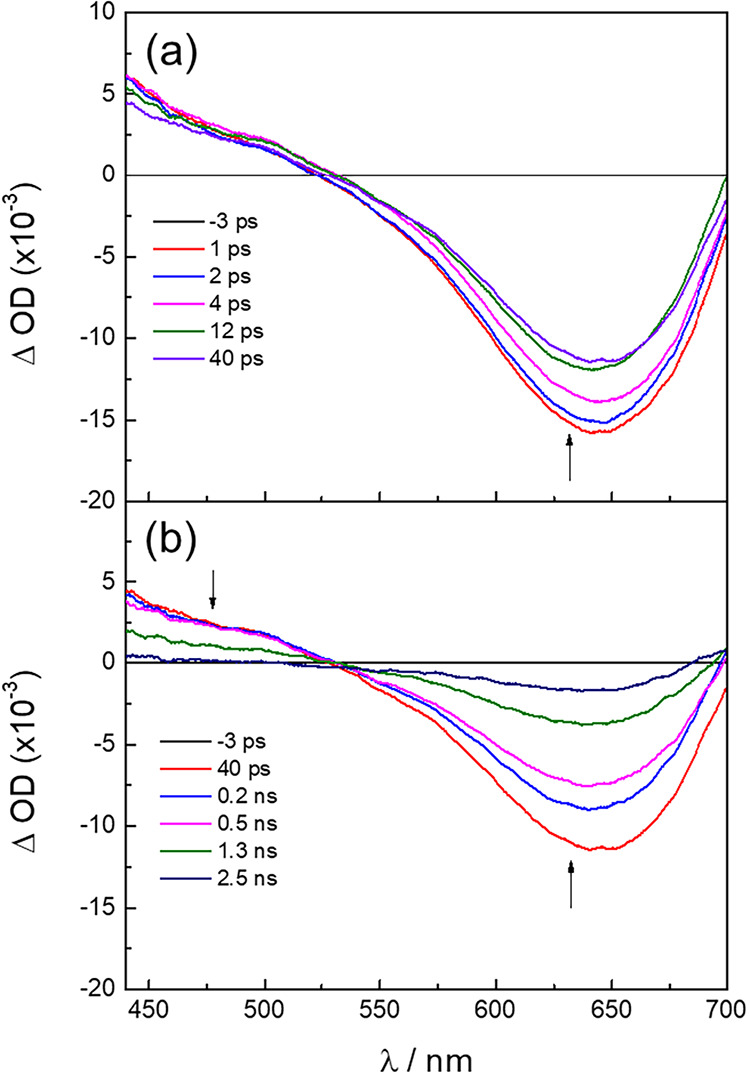
Ultrafast transient absorption spectra of **1** collected at (a) early and (b) later delay times after the excitation pulse in CH_3_CN (λ_exc_ = 400 nm, FWHM = 85 fs, 298 K).

The nsTA spectra measured for **6** are shown in Figure 5 (λ_exc_ = 600 nm, IRF = 7 ns) and possess similar spectral features to those recorded in the fsTA experiment (Figure S8d). Excited state absorption peaks associated with the ^3^ML-LCT state are observed at 380 and 425 nm, along with a bleach signal from 525 to 675 nm, both of which decay monoexponentially with τ_T_ = 54 ns. This lifetime represents the longest recorded to date for a ^3^ML-LCT excited state of a dirhodium­(II,II) complex.^12,31^ Shorter ^3^ML-LCT lifetimes were measured for **2** (11 ns), **4** (17 ns), and **5** (22 ns), while that of **3** was previously reported, τ_T_ = 19 ns.^11^ The lengthening of the triplet excited state lifetime from **1** to **6** follows the corresponding increase in E_00_^T^ (Table 2), showing that the series obeys the Energy Gap Law, whereby nonradiative deactivation is slower for complexes with greater separation between the ^3^ML-LCT and the ground state.

**Figure 5:**
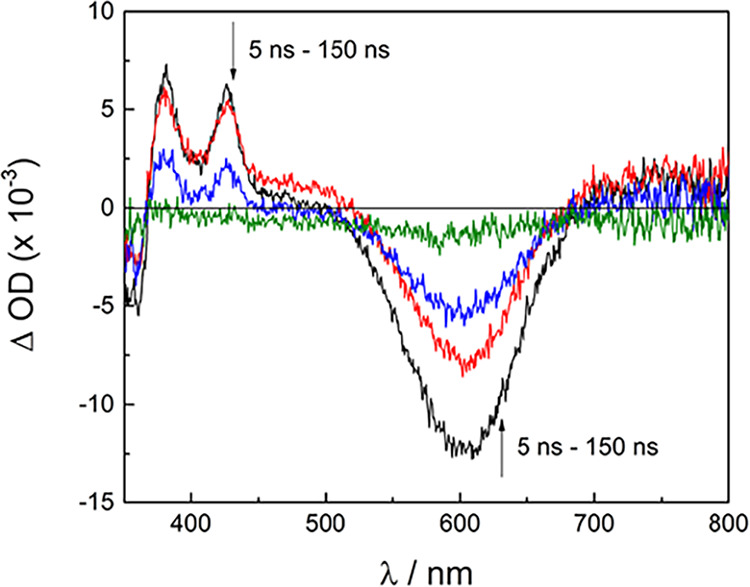
Nanosecond transient absorption spectra of **6** collected 5, 10, 50, and 150 ns after the 600 nm (FWHM = 7 ns) excitation pulse in CH_3_CN at room temperature.

### Photocatalytic H_2_ Production

The photocatalytic cycle for H_2_ production in this class of Rh_2_(II,II) complexes is shown in Figure 6, which starts with the excitation of [Rh_2_] and its reductive quenching by the sacrificial electron donor BNAH to generate [Rh_2_]^−^. In previous work, the singly reduced complex [Rh_2_]^−^ has been shown to be photoactive and to generate the doubly reduced species, [Rh_2_]^2–^, under irradiation in the presence of BNAH.^11,12^ The singly and doubly reduced complexes have been isolated following chemical and electrochemical treatment. Both [Rh_2_]^−^ and [Rh_2_]^2–^ are able to produce hydrogen in the presence of TsOH in the dark, leading to the two pathways for H_2_ evolution depicted in Figure 6, *Path 2* and *Path 1*, respectively. The minor pathway in the catalytic cycle, *Path 2*, involves the protonation of [Rh_2_]^−^ to generate [Rh_2_]–H, followed by the reaction of two [Rh_2_]–H molecules to produce H_2_ and regenerate two equivalents of [Rh_2_] (Figure 6). The major pathway, *Path 1*, involves the generation of [Rh_2_]^2–^ through the reductive quenching of *­[Rh_2_]^−^ by BNAH, which reacts with acid to produce H_2_ and [Rh_2_] (Figure 6).^11,12^

**Figure 6:**
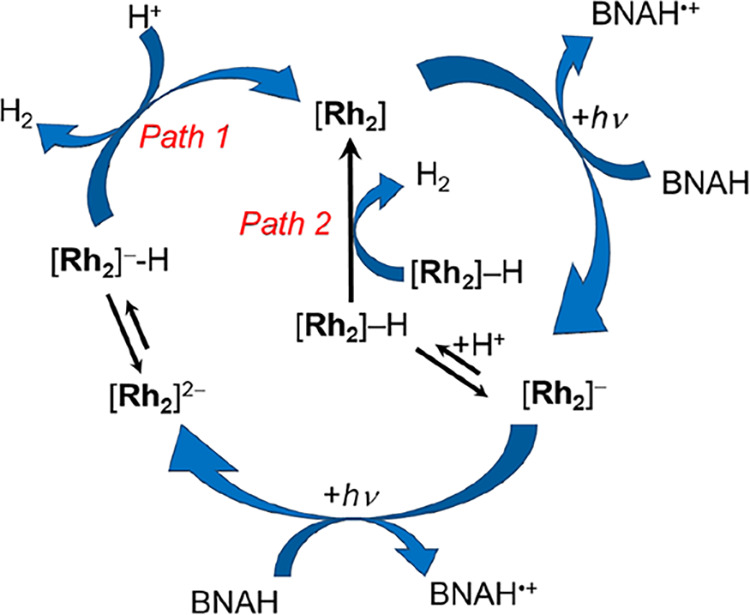
Proposed photocatalytic cycle for H_2_ evolution from **3** and related complexes.

The turnover numbers (TONs) for H_2_ production measured for **1**–**6** after 24 h of 595 nm irradiation in the presence of 0.1 M TsOH and 30 mM BNAH in DMF are summarized in Table 2, along with the ^3^ML-LCT excited state reduction potential, ^3^E*_red_, and the driving force for the reductive quenching by BNAH, Δ*G*_RQ_, for each complex. The highest TON, 125, is observed for **3**, with τ_T_ = 19 ns and Δ*G*_RQ_ = −0.38 V, while **1** and **6** display the lowest values, TON = 12. Recent work using related complexes has shown that quenching of the excited state, *­[Rh_2_], by BNAH is the first step following photon absorption in the catalytic cycle (Figure 6), and that the excited state lifetime plays a prominent role in the overall reactivity.^12,31^ For example, in *cis*-[Rh_2_(OAc)­(DPhF)­(bncn)_2_]^2+^ (**7**), with τ_T_ = 2.7 ns, ^3^E*_red_ = +1.26 V vs Ag/AgCl, and Δ*G*_RQ_ = −0.65, a considerably lower TON value was observed in comparison to **3** under similar experimental conditions.^12b^ The ^3^E*_red_ is greater for **7** than that of **3**, +0.99 V vs Ag/AgCl (Table 2), indicating that the driving force for the formation of the radical anion intermediate is not the reason for the lower reactivity of the former. In addition, the photocatalytic activity of **7** was shown to increase with BNAH concentration, consistent with the lower level of reductive quenching to produce the required intermediate, [**7**]^−^, afforded by its shorter excited state lifetime.^12b^ Complexes **1**, **2**, and **4** exhibit shorter excited state lifetimes and similar ^3^E*_red_ values relative to **3**, and the TONs of the former are lower than that of the latter (Table 2), in agreement with the key role of the excited state lifetime of the complex in H_2_ production. This finding indicates that the quenching of the excited state to generate the singly reduced species, [Rh_2_]^−^, is the rate-limiting step in the photocatalytic cycle.^11,12^ In order to further ascertain this point, identical concentrations of **3** and **6** were electrochemically reduced in the presence of 0.1 M TsOH, which resulted in greater electrocatalytic current for **6** (Figure S9). These experiments rule out the reaction of [Rh_2_]^2–^ with protons as the rate-limiting step in **6**. Importantly, while **5** and **6** exhibit longer τ_T_ values and more favorable Δ*G*_RQ_ than **3**, lower TON values were measured for these complexes (Table 2). Since the reductive quenching of **6** to produce [**6**]^−^ and the reaction of [**6**]^2–^ with protons are not limiting the H_2_ production, it is possible that in **6** the rate-limiting step may be associated with the reactivity of [Rh_2_]^−^.

In order to investigate the reactivity of [**1**]^−^–[**6**]^−^, DFT calculations and time-resolved experiments were conducted. The energies and characters of the highest singly occupied and highest doubly occupied molecular orbitals, the SOMO and HDOMO, respectively, as well as those of the four lowest unoccupied orbitals, the LUMO–LUMO + 3, are listed in Table S2. As an example, the calculated electron densities of these frontier molecular orbitals for [**3**]^−^ are displayed in Figure 7. The SOMOs in [**1**]^−^–[**6**]^−^ are localized on a π* orbital on the bncn^–^ ligand, as shown for [**3**]^−^ in Figure 7, and are slightly stabilized by 0.16 eV across the series. This trend is expected from the results for the corresponding complexes **1**–**6**, with modest stabilization of the calculated LUMO and the small anodic shift of 0.09 V in the first reduction couple, *E*_1/2_(bncn)^0/–^, from **1** to **6** (Table 1). The HDOMO features Rh_2_(δ*)/Form­(π/nb) character, as depicted for [**3**]^−^ in Figure 7, and is stabilized by 0.48 eV from [**1**]^−^ to [**6**]^−^, similar to the 0.53 eV stabilization calculated for the HOMOs of **1**–**6** (Figure S4). The nondegenerate LUMO and LUMO+1 for [**1**]^−^–[**6**]^−^ are both calculated to be localized on neutral bncn^0^ π* orbitals, shown for [**3**]^−^ in Figure 7. Across the series, the LUMO + 1 lies at ∼1.3 eV higher in energy than the respective LUMO in each complex. The LUMO is stabilized by 0.16 eV from [**1**]^−^ to [**6**]^−^, and the LUMO+1 by 0.09 eV, similar to the trend observed for the SOMOs in the series. The LUMO+2 MOs of [**1**]^−^–[**6**]^−^ possess ∼70% Rh_2_(σ*) character involving the d_*z*2_ orbitals, while the LUMO+3 exhibits ∼50% Rh_2_-L­(σ*) d_*x*2_–_*y*2_ character (see Figure 7 for [**3**]^−^). The stabilization of the LUMO + 2 and LUMO + 3 was calculated to be considerably larger from [**1**]^−^ to [**6**]^−^, 0.28 and 0.43 eV, respectively, in agreement with the theoretical and the electrochemical observations for **1** to **6** (Table 1 and Figure S4).

**Figure 7:**
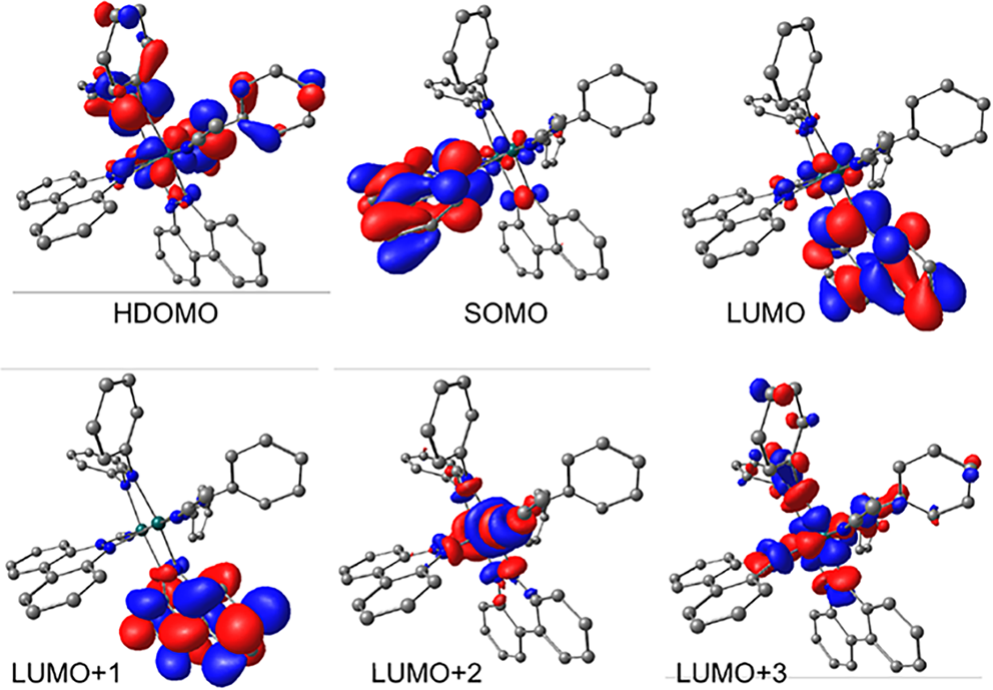
Electron density of the calculated HDOMO to LUMO+3 for [**3**]^−^ (plotted at 0.2 isovalue; hydrogen atoms are omitted for clarity).

The geometry optimization of each [Rh_2_]^**–**^ anion radical resulted in two types of bncn ligands, one neutral and the other reduced, denoted herein as bncn^0^ and bncn^–^, respectively. The N–N bond distance in bncn^–^ calculated for [**3**]^−^, 1.357 Å, is longer than that of bncn^0^, 1.305 Å, as expected from the presence of an occupied π* MO in the former. The latter is in agreement with the bncn N–N distance of 1.308 Å observed in the X-ray crystal structure and the 1.301 Å calculated for **3**. A similar trend is observed for the remaining [Rh_2_]^−^ complexes in the series, where the bncn^–^ ligands exhibit ∼0.05 Å longer N–N bond lengths than bncn^0^ in each complex.

TD-DFT calculations were performed for [**1**]^−^–[**6**]^−^, and the energies of the resulting doublet excited states are displayed in Figure 8. The lowest energy excited states in [**1**]^−^ to [**6**]^−^ were calculated to be bncn­(π*)^−^ → bncn­(π*) ligand-to-ligand charge transfer (^2^LLCT) in nature and to lie ∼0.24 eV above the doublet ground state, ^2^GS. This result suggests that the placement of the electron on one or the other bncn ligand in the reduced complexes is in equilibrium at room temperature, consistent with the degenerate LUMO and LUMO + 1 calculated for **1**–**6**. Therefore, the contribution of the ^2^LLCT excited state to the photophysical properties of the [Rh_2_]^−^ complexes can be ruled out. The lowest energy excited state above the ^2^LLCT is calculated to be a Rh_2_(δ*)/Form­(π/nb) → bncn­(π*) ^2^ML-LCT state for [**1**]^−^–[**3**]^−^ and a bncn­(π*)^−^ → Rh_2_(σ*) ligand-to-metal charge transfer (^2^LMCT) for [**4**]^−^–[**6**]^−^. The energy of the ^2^ML-LCT state increases slightly across the series, from 1.40 eV in [**1**]^−^ to 1.70 eV in [**6**]^−^ above the ^2^GS, while the ^2^LMCT state is stabilized from [**2**]^−^–[**5**]^−^, falling below the ^2^ML-LCT state in [**4**]^−^–[**6**]^−^ (Figure 8). In the case of [**4**]^−^–[**6**]^−^, a low lying bncn­(π*)^−^ → Rh_2_-L­(σ*) ligand-to-metal/ligand charge transfer (^2^L-MLCT) state is also calculated (Figure 8).

**Figure 8:**
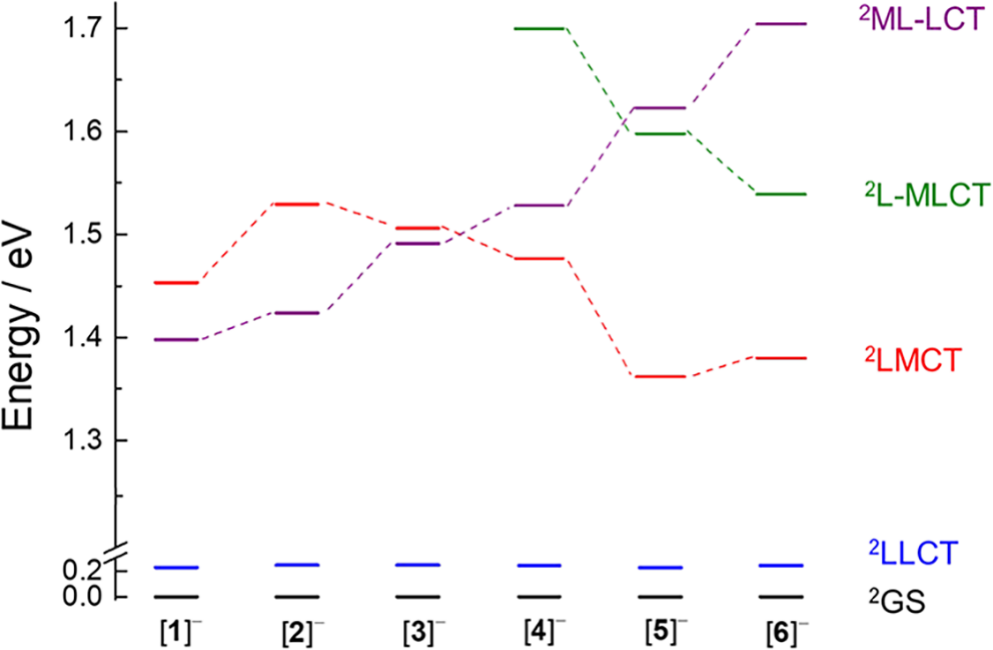
Calculated vertical energies of the ^2^LLCT (blue), the ^2^LMCT (red), the ^2^L-MLCT (green), and the ^2^ML-LCT (purple) excited states and the ground state, ^2^GS (black), of [**1**]^−^–[**6**]^−^ in CH_3_CN.

Transient absorption spectra were collected for [**2**]^−^, [**3**]^−^, [**5**]^−^, and [**6**]^−^, and their steady-state absorption spectra in CH_3_CN are shown in Figure S10, with the absorption maxima and the respective doublet excited state lifetimes, τ_D_, listed in Table 3. The fsTA spectra measured for [**2**]^−^ and [**6**]^−^ in CH_3_CN are shown in Figure 9, while those of [**3**]^−^ and [**5**]^−^ are displayed in Figure S11 (λ_exc_ = 650 nm, FWHM = 85 fs). A broad excited state absorption is observed for [**2**]^−^ with two sharp peaks at 424 and 445 nm, along with a broader positive signal at 530 nm (Figure S11a). The decay of the 424 nm band was fitted using a biexponential function, resulting in lifetimes of 3 ps and 1.2 ns. This feature is similar to the excited state absorption observed for **1**–**6**, and can be assigned to the absorption of reduced bncn in the ^2^ML-LCT state with τ_D_ = 1.2 ns.^11,12^ This finding is consistent with the TD-DFT calculations, where the ^2^ML-LCT state is the lowest energy excited state at room temperature in this complex (Figure 8). The short component, 3 ps, can be assigned as arising from vibrational cooling in the ^2^ML-LCT excited state.

**Figure 9:**
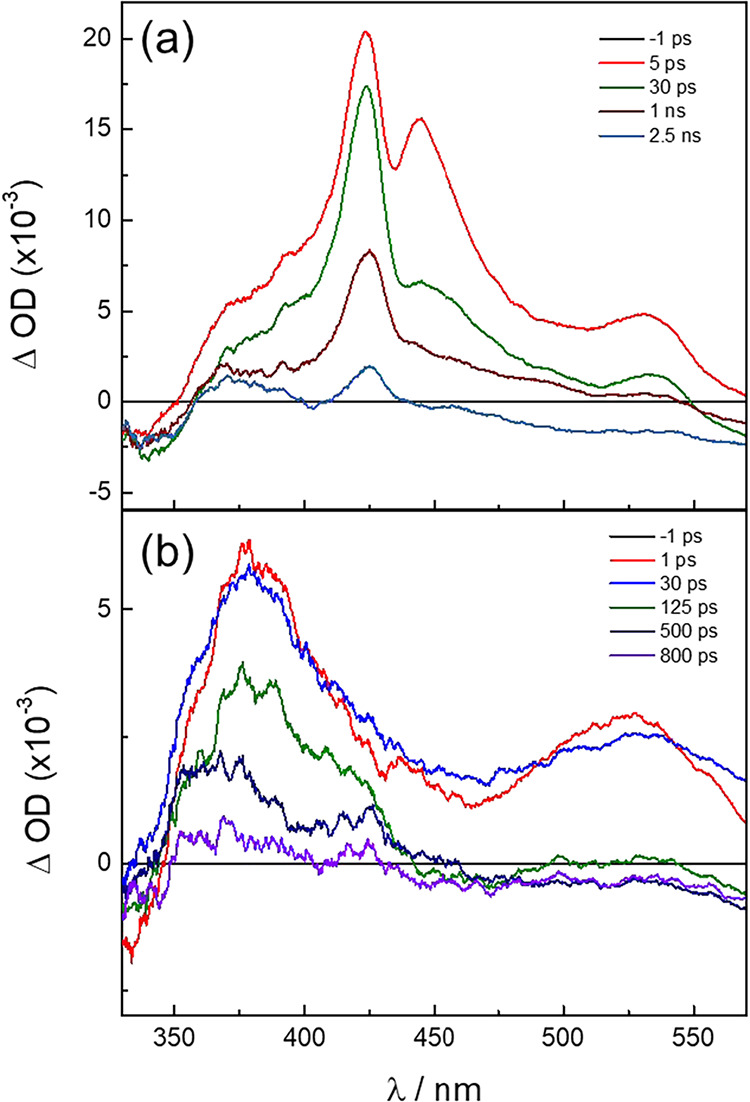
Ultrafast transient absorption spectra of (a) [**2**]^**–**^ and (b) [**6**]^**–**^ collected after the excitation pulse in CH_3_CN (λ_exc_ = 650 nm, FWHM = 85 fs, 298 K).

**Table 3:** Electronic Absorption Maxima of the ^2^ML-LCT Transition (λ_max_), Molar Extinction Coefficients, ε, and Doublet Excited State Lifetimes, τ_D_, of [**2**]^**–**^, [**3**]^**–**^, [**5**]^**–**^, and [**6**]^**–**^ in CH_3_CN

		ε × 10^3^ M^–1^ cm^–1^		
complex	λ_max_/nm	λ_max_	λ_irr_ ^a^	τ_D_/ns
[**2**]^−^	682	5.6	3.9	1.2
[**3**]^−^	671	5.0	3.7	0.49
[**5**]^−^	654	4.9	3.8	0.43
[**6**]^−^	628	5.9	5.1	0.26

a Irradiation wavelength = 595 nm.

The 445 nm excited state absorption decays to the ground state monoexponentially with a lifetime of 15 ps and is attributed to an intraligand transition localized on the bncn^–^ ligand in [**2**]^−^. Previous work has shown that the free bncn^–^ ligand displays a broad absorption throughout the visible range, with maxima at 400 nm, 440 nm and ∼650 nm.^12b^ Since the spectra in Figure 9 were recorded following 650 nm excitation, a wavelength where bncn^–^ absorbs strongly, we have assigned this signal as arising from an excited state absorption localized on the bncn^–^ radical anion bound to the dirhodium core.^15–17^ The presence of two signals with different dynamics was reported in the fsTA spectra of [Ir­(dFCF_3_ppy)_2_(R_2_-bpy^•–^)] with 350 nm excitation, one set with a biexponential decay with lifetimes ∼7 ps and ∼18 ps, and the other, a broad signal at ∼475 nm, decayed monexponentially with τ ∼ 15 ps.^13^ Importantly, the positive feature at ∼475 nm was not present with λ_exc_ = 800 nm.^13^ In this singly reduced complex, the electron is localized on the R_2_-bpy ligand; the bpy^•–^ radical anion is known to absorb strongly at 350 nm and weakly, if at all, at 800 nm in the free ligand and in [Ru­(bpy)_2_(bpy^•–^)]^+^.^15,37^ Since the 15 ps signal in the 450–500 nm range is only present with 350 nm excitation, it can be assigned as arising from a ligand-centered excited state associated with the coordinated R_2_-bpy^•–^ in [Ir­(dFCF_3_ppy)_2_(R_2_-bpy^•–^)]. These observations of the TA spectra of [Ir­(dFCF_3_ppy)_2_(R_2_-bpy^•–^)] support the assignment herein of the 445 nm signal, which also decays with a 15 ps lifetime, to transitions localized on the bncn^•–^ ligand excited at a wavelength where the bncn anion is known to absorb strongly.

Similar fsTA spectral features are observed for [**3**]^−^ and [**5**]^−^, where the positive signal associated with the ^2^ML-LCT state decays with 0.49 and 0.43 ns components, respectively, and the intraligand bncn^–^ excited state with τ < 25 ps. The decay of the band at 420 nm observed in the TA spectra of [**3**]^−^ (Figure S11a) was fitted using a biexponential function with 1 ps and 0.49 ns components, while the 444 nm feature corresponding to the intraligand bncn^–^ transition decayed more rapidly, with a lifetime of 23 ps, similar to [**2**]^−^. In the fsTA spectra of [**5**]^−^ (Figure S11b), the bands at 424 and 444 nm decayed monoexponentially with lifetimes of 0.43 ns and 11 ps, respectively.

The fsTA spectra of [**6**]^−^ are composed of a broad excited state absorption with peaks at 380 and 530 nm, both of which decay monoexponentially with a lifetime of 260 ps. The difference in the excited state absorption spectral profiles observed for [**6**]^−^ as compared to those recorded for [**2**]^−^–[**5**]^−^ suggests that a different character of the lowest energy excited in the former. This finding is in agreement with the theoretical calculations, where a ^2^LMCT lowest energy excited state is calculated for [**6**]^−^ (Figure 8). It is interesting to note that the bncn^–^-centered peak at 445 nm with τ ∼ 15 ps is not observed in the fsTA of [**6**]^−^. It is hypothesized that this ligand-localized state decays within the laser pulse to the lowest energy ^2^LMCT state in [**6**]^−^, whereas its internal conversion to the ^2^ML-LCT states in [**2**]^−^–[**5**]^−^ is slower, allowing its observation in the present pump–probe experiments with 85 fs pulses. While the spectral differences in the TA between [**2**]^−^ and [**6**]^−^ can be explained by the nature of the calculated lowest energy excited state in these complexes, the energy of the ^2^MLCT state of [**5**]^−^ is also calculated to be stabilized relative to the ^2^ML-LCT state. This is a point that will require additional clarification in future work.

The excited state lifetimes, τ_D_, decrease from 490 ps in [**3**]^−^, to 430 ps in [**5**]^−^, to 260 ps in [**6**]^−^. The shorter lifetime of [**6**]^−^ is expected to decrease the amount of bimolecular reductive quenching of the excited state to form the reactive [**6**]^2**–**^ (Figure 6). This trend helps explain the lower photocatalytic H_2_ production in **6** relative to **3** and **5**, and shows that the reductive quenching of [Rh_2_]^−^ may be the rate-limiting step in *Path 1* for the photocatalytic cycle of **6**. Alternatively, owing to the short excited state lifetime of [**6**]^−^, it is possible that *Path 1* is no longer operative and this complex relies on *Path 2* for photocatalysis (Figure 6). It should be noted, however, the role of the change in excited state character from ^2^ML-LCT in [**3**]^−^ to ^2^LMCT in [**6**]^−^ in the excited state reactivity cannot be ruled out at this time. Previous work with **3** and related complexes has shown that *Path 1* in Figure 6 is the major pathway for photocatalytic H_2_ evolution and that *Path 2*, while operative, is minor.^11,12a^ Therefore, complexes with shorter-lived *­[Rh_2_] and *­[Rh_2_]^−^ excited states are expected to exhibit less efficient *Path 1* in catalytic mixtures containing identical concentrations of Rh_2_(II,II) complex, acid, and sacrificial electron donor, making *Path 2* a viable route for H_2_ generation in these systems (Figure 6). In *Path 1*, the formation of the [Rh_2_]^2**–**^ species from the reductive quenching of *­[Rh_2_]^−^ represents a critical step for H_2_ production, which is limited by the lifetime of the excited state of *­[Rh_2_]^−^. In contrast, *Path 2* relies on the ground-state protonation of [Rh_2_]^−^ to generate [Rh_2_]-H. The bimolecular reaction of two [Rh_2_]-H species in solution generates H_2_ and two equivalents of the starting material, [Rh_2_].

In order to explore the reactivity of [**6**]^−^ with acid to regenerate **6**, the recovery of the ^1^ML-LCT absorption of **6** was monitored as a function of time following the addition of excess TsOH to a solution of [**6**]^−^, and the result is shown in Figure S12. The experiment with identical concentrations of [**3**]^−^ and TsOH shows that the rate of reaction is faster for [**6**]^**–**^ as compared to [**3**]^**–**^ (Figure S12), consistent with the hypothesis that *Path 2* is more efficient in the former relative to the latter. In the presence of 30 mM BNAH, but in the absence of acid, the ML-LCT absorption band of [**6**]^−^ decreases in intensity upon irradiation as [**6**]^2**–**^ accumulates in the solution (Figure S13a). Similar spectral changes have been previously reported for **3** and are shown in Figure S13b under similar conditions.^11^ However, in the presence of excess acid and BNAH, the protonation of [**6**]^**–**^ following the reductive quenching of **6**, results in a new feature at 690 nm, believed to be [**6**–H] (Figure S13c). In contrast, the spectral changes observed for **3** under similar conditions include a broad absorption with a maximum at ∼640 nm and a shoulder at ∼755 nm. The former feature is an indication of the regeneration of **3** in the catalytic solution along with another species, which can be attributed to the doubly reduced, singly protonated complex, [**3**–H]^−^ (Figure S13d). These results indicate that different species are accumulated upon irradiation of the catalytic mixtures of **3** and **6**, with the regeneration of **3** indicating that the catalyst gets back to its initial state after completing the photocatalytic cycle, while the process is not completed in **6** under similar experimental conditions.

## Conclusions

A series of heteroleptic [Rh_2_] complexes, *cis-*[Rh_2_(*p-*R-Form)_2_(bncn)_2_]^2+^, where R = −OCH_3_ (**1**), −CH_3_ (**2**), −H (**3**), −F (**4**), −Cl (**5**), and −CF_3_ (**6**), was synthesized and chemically reduced to obtain the corresponding singly reduced species, [Rh_2_]^−^, believed to be important intermediates in the photocatalytic production of H_2_. The photophysical and redox properties of **1**–**6** and [**1**]^−^–[**6**]^−^were investigated to help elucidate the photocatalytic mechanism for H_2_ evolution and the observed differences in TONs across the series. The stabilization of the Rh_2_(δ*)/DPhF­(π/nb) HOMO is observed from **1** to **6**, where the [Rh_2_]^3+/2+^ couple shifts anodically by 370 mV. In contrast, the bncn­(π*)-centered LUMO displays a relatively smaller anodic shift of 90 mV across the series. The HOMO → LUMO ^1^ML-LCT transition shifts to higher energy, from 656 nm in **1** to 599 nm in **6** in CH_3_CN, consistent with the increase in the experimental and calculated HOMO–LUMO gaps and the energy of the ^1^ML-LCT states obtained from TD-DFT across the series. The ^3^ML-LCT energy, E_00_^T^, also increases from **1** to **6**, resulting in more favorable values for the driving force for the reductive quenching by BNAH, Δ*G*_RQ_. Complex **6** displays the longest excited state lifetime, τ_T_ = 54 ns, while the shortest was recorded for **1**, 1.6 ns, consistent with the Energy Gap Law. Although **5** and **6** exhibit longer τ_T_ values and more negative ΔG_*RQ*_ than the other complexes in the series, the photocatalytic H_2_ evolution upon irradiation (λ_irr_ = 595 nm) of [Rh_2_] solutions in the presence of BNAH and TsOH, was observed to be the largest for **3** (TON = 125), while **5** and **6** displayed TONs of 62 and 12, respectively.

Additional theoretical and experimental studies of the anionic species, [Rh_2_]^−^, were undertaken to understand the limiting step in the photocatalytic cycle of **6**. TD-DFT showed that the lowest energy excited state changed from ^2^ML-LCT in [**1**]^−^–[**3**]^−^ to ^2^LMCT in [**4**]^−^–[**6**]^−^. Ultrafast TA spectra of [**2**]^−^, [**3**]^−^ and [**5**]^−^ (λ_ex_ = 650 nm) displayed three excited state absorption bands, where the peaks at ∼422 nm and ∼444 nm were assigned to the ^2^ML-LCT and intraligand bncn^–^-centered excited states, respectively. The TA spectra of [**6**]^−^ displayed excited state absorption maxima at 380 and 530 nm. The different spectral features observed for [**6**]^−^ are consistent with the theoretical calculations, indicating that the lowest energy excited state is ^2^LMCT and not ^2^ML-LCT. The doublet excited state lifetimes, τ_D_, for [**2**]^−^, [**3**]^−^, [**5**]^−^, and [**6**]^−^ were measured to be 1.2 ns, 0.49 ns, 0.43 ns, and 0.26 ns, respectively. Complex **3** and related complexes have been previously shown to follow *Path 1* (Figure 6) as the major pathway for H_2_ evolution, where the formation of the [Rh_2_]^2–^ intermediate from the photoinduced reductive quenching of [Rh_2_]^−^ is a key step. The shorter τ_D_ of [**6**]^−^ renders the reductive quenching of the excited state and generation of [**6**]^2–^ kinetically challenging as compared to [**3**]^−^. Photolysis of solutions of **6** and BNAH and TsOH in DMF also shows the accumulation of an intermediate that is different than that observed under identical conditions of **3**. It is hypothesized that [**6**-H] may be accumulating upon irradiation of catalytic mixtures containing **6**, whereas the regeneration of the starting material and possibly [**3**-H]^−^ is observed for **3**. This result indicates that *Path 2* may be the major route for photocatalytic hydrogen production by **6**. These findings highlight the importance of the radical anion intermediate for photocatalytic generation of H_2_ using dirhodium­(II,II) complexes and provide useful information for the use of earth-abundant metal complexes containing bncn ligands in the future.

## Supplementary Material



## Notes

Crystal structures, emission and excitation spectra of **1**, **2**, and **4–6**, fsTA of **2**, **4**, **5**, **6**, and [**3**]^**–**^ and [**5**]^**–**^ in CH_3_CN, catalytic currents observed for **3** and **6**, spectral changes upon photolysis of catalytic mixtures of **3** and **6**, absorption changes upon reaction of [**3**]^**–**^ and [**6**]^**–**^ with TsOH, additional computational data for [**Rh**_**2**_] and [**Rh**_**2**_]^−^ complexes, variation of E_00_^T^ and *E*_1/2_[Rh_2_]^3+/2+^ with 4σ_R_ for **1–6**, and crystallographic details (PDF)
