# Supplementary material for: Role of the Character of the Excited State of Singly Reduced Rh2(II,II) Intermediates on Photocatalytic Activity
Source: J Am Chem Soc. 2026 Apr 15;148(16):17197–207. doi: 10.1021/jacs.6c02339 (PMC13133775; doi:10.1021/jacs.6c02339)
Supplement: Supplementary file 1 [file ja6c02339_si_001.pdf]

Supporting Information

Role of the Character of the Excited State of Singly-Reduced Rh_2(II,II) Intermediates on Photocatalytic Activity

Piyush Gupta, Curtis E. Moore, and Claudia Turro*

Department of Chemistry and Biochemistry, The Ohio State University, Columbus, Ohio 43214, USA

Table of Contents **Page Number**

Synthetic scheme for **1** – **6** S1

Cystal Structures of **1, 2** and **4** – **6** S3

$E_{1/2}$[$\mathbf{Rh_2}$]$^{3+/2+}$ *vs* 4σ_R for **1** – **6** S3

CV for **1** in CH_3CN S4

Table S1 .... S4

Calculated MO diagrams for **1** – **6** S5

Electron density on HOMO and LUMO for **1** – **6**.. S6

Emission at 77 K for **1**, **2** and **4** – **6**.. S7

^{3}ML-LCT energies *vs* 4σ_R value for **1** – **6** S8

fsTA of **2** and **4** – **6** in CH_3CN S8

Catalytic currents observed for **3** and **6** in presence of TsOH.. S9

Absorption spectra of [**2**]$^-$, [**3**]$^-$, [**5**]$^-$, and [**6**]$^-$ in CH_3CN.. S9

fsTA of [**3**]$^-$ and [**5**]$^-$ in CH_3CN.. S10

Table S2 .... S10

Recovery of ^{1}ML-LCT absorption upon reaction of TsOH with [**3**]$^-$ and [**6**]$^-$.. S11

Spectral changes upon photolysis of **3** and **6** and BNAH in presence and absence of acid...... S11

Summary of crystallographic data.. S12–S38

General synthetic procedure for *cis*-$Rh_2(OAc)_2$(*p*-R-Form)$_2$ (1a, 2a, 4a, 5a, 6a). $Rh_2(\mu\text{–}OAc)_4$ (50 mg, 0.113 mmol) and 2 eq of *p*-R-HForm (R = OMe, Me, F, Cl, CF_3) were suspended in 7 mL of dichloroethane in a 10 mL microwave vessel and sealed with the cap. The suspension was stirred for one minute and then heated at 190 °C (200 W) for 10 minutes in a microwave reactor. The resulting dark green solution was dried under vacuum, redissolved in acetone and passed through a silica column using 70:30 (hexane:acetone, v:v) elusion. The product was obtained as a green powder upon removal of solvent under vacuum.

1a (R= OMe). Yield: 52 mg (55%). ^{1}HNMR in CD_2Cl_2 400 MHz: ppm (mult., *J*, int): 7.38 (*t*, 4.0 Hz, 2H), 6.94 (*d*, 8.0 Hz, 8H), 6.72 (*d,* 8.0 Hz, 8H), 2.04 (*s*, 6H).

2a (R = CH_3). Yield: 43 mg (60%). ^{1}HNMR in CD_2Cl_2 400 MHz: ppm (mult., *J*, int): 7.47 (*t*, 4.0 Hz, 2H), 6.98 (*m*, 16H), 2.26 (*s*, 12H), 2.01 (*s*, 6H).

4a (R = F). Yield: 57 mg (65%). ^{1}HNMR in CD_2Cl_2 400 MHz: ppm (mult., *J*, int): 7.42 (*s*, 2H), 6.97 (*t,* 7.0 Hz, 8H), 6.88 (*t,* 7.0 Hz, 8H), 2.04 (*s*, 6H).

5a (R = Cl). Yield: 58 mg (61%). ^{1}HNMR in CD_2Cl_2 400 MHz: ppm (mult., *J*, int): 7.45 (*t,* 4.0 Hz, 2H), 7.15 (*d,* 8.0 Hz, 8H), 6.95 (*d,* 8.0 Hz, 11H), 2.05 (*s*, 6H).

6a (R = CF_3). Yield: 67 mg (60%). ^{1}HNMR in CD_2Cl_2 400 MHz: ppm (mult., *J*, int): 7.63 (*s*, 2H), 7.43 (*d*, 8.0 Hz, 8H), 7.15 (*d*, 8.0 Hz, 8H), 2.05 (*s*, 3H).

Gneral synthetic procedure for *cis*-[Rh_2(*p*-R-Form)$_2$(CH_3CN)$_6$][BF_4]$_2$ (1b, 2b, 4b, 5b, 6b). *Cis*-$Rh_2(OAc)_2$(*p*-R-Form)$_2$ (35 mg, ~0.04 mmol) was dissolved in 10 mL of CH_2Cl_2/CH_3CN (1:1, v:v) solution and heated to 45 °C, followed by addition of 8 eq of Et_3OBF_4. The solution was stirred at the same temperature overnight under N_2 atmosphere. The reaction mixture was then cooled down and the solvent was removed under reduced pressure. The remaining solid was redissolved in minimal amount (<1 mL) of acetonitrile and was added dropwise to 50 mL of diethyl ether which was stirred for one hour and then filtered over a medium coarse frit. A gray paste was obtained on the frit which was further washed with 2 x 30 mL of diethyl ether. The final product was collected upon redissolving the gray paste in acetonitrile and removing the solvent under a stream of N_2 to get a red powder.

1b (R = OMe). Yield: 43 mg (96%). ^{1}HNMR in CD_3CN 400 MHz: ppm (mult., *J*, int): 7.45 (*t*, 4.0 Hz, 2H), 7.03 (*d*, 8.0 Hz, *d*, 8H), 6.79 (*d*, 8.0 Hz, 8H).

2b (R = CH_3). Yield: 46 mg (93%). ^{1}HNMR in CD_3CN 400 MHz: ppm (mult., *J*, int): 7.38 (*t*, 4.0 Hz, 2H), 6.94 (*q*, 8.0 Hz, 16H).

4b (R = F). Yield: 40 mg (80%). ^{1}HNMR in CD_3CN 400 MHz: ppm (mult., *J*, int): 7.51 (*t*, 4.0 Hz, 2H), 7.12 – 7.08 (*m*, 8H), 6.99 (*t*, 8.0 Hz, 8H).

5b (R = Cl). Yield: 48 mg (99%). ^{1}HNMR in CD_3CN 400 MHz: ppm (mult., *J*, int): 7.56 (4.0 Hz, *t*, 2H), 7.25 (8.0 Hz, *dt*, 8H), 7.09 (8.0 Hz, *dt*, 8H).

6b (R = CF_3). Yield: 40 mg (88%). ^{1}HNMR in CD_3CN 400 MHz: ppm (mult., *J*, int): 7.80 (*t*, 4.0 Hz, 2H), 7.61 (*d*, 8.0 Hz, 8H), 7.34 (*d*, 8.0 Hz, 8H).

General synthetic procedure for *cis*-[Rh_2(*p*-R-Form)$_2$(bncn)$_2$][BF_4]$_2$ (1, 2, 4, 5, 6). *Cis*-[Rh_2(*p*-R-Form)$_2$(CH_3CN)$_6$][BF_4]$_2$ (40 mg, ~0.03 mmol) and 3 eq of bncn were stirred in 20 mL CH_2Cl_2 in a 4 dram vial at room temperature for 15 minutes upon which the color of the solution changed from bright green to deep blue. The solution was dried under a stream of N_2, redissolved in minimum amount of acetonitrile and washed with diethyl ether several times to remove any excess ligand.

1 (R = OMe). Yield: 39 mg (94%). ^{1}HNMR in CD_3CN, 400 MHz: ppm (mult., *J*, int): 9.66 (*dd*, 8.0 Hz, 4H), 8.81 (*dd*, 8.0 Hz, 4H), 8.24 (*t*, 8.0 Hz, 4H), 8.19 (*t*, 8.0 Hz, 4H), 6.93 (*t,* 4.0 Hz, 2H), 6.80 (*q,* 8.0 Hz, 8H), 3.77 (*s,* 12H).

2 (R = CH_3). Yield: 42 mg (95%). ^{1}HNMR in CD_3CN, 400 MHz: ppm (mult., *J*, int): 9.66 (*dd*, 8.0 Hz, 4H), 8.81 (*dd*, 8.0 Hz, 4H), 8.22 (*dq*, 8.0 Hz, 8H), 7.03 (*d*, 8.0 Hz, 8H), 6.95 (*t,* 4.0 Hz, 2H), 6.80 (*d,* 8.0 Hz, 8H), 2.28 (*s,* 12H).

4 (R = OMe). Yield: 44 mg (99%). ^{1}HNMR in CD_3CN, 400 MHz: ppm (mult., *J*, int): 9.66 (*d*, 8.0 Hz, 4H), 8.82 (*d*, 8.0 Hz, 4H), 8.29 (*dt*, 8.0 Hz, 4H), 8.21 (*dt,* 8.0 Hz, 4H), 7.03 (*t,* 8.0 Hz, 8H), 6.95 (*t,* 4.0 Hz, 2H), 6.93-6.89 (*m,* 8H).

5 (R = OMe). Yield: 43 mg (99%). ^{1}HNMR in CD_3CN, 400 MHz: ppm (mult., *J*, int): 9.64 (*d*, 8.0 Hz, 4H), 8.83 (*d*, 8.0 Hz, 4H), 8.29 (*dt*, 8.0 Hz, 4H), 8.22 (*dt,* 8.0 Hz, 4H), 7.27 (*d*, 8.0 Hz, 8H), 6.94 (*t,* 4.0 Hz, 2H), 6.89 (*d,* 8.0 Hz, 8H).

6 (R = OMe). Yield: 43 mg (99%). ^{1}HNMR in CD_3CN, 400 MHz: ppm (mult., *J*, int): 9.66 (*d*, 8.0 Hz, 4H), 8.87 (*d*, 8.0 Hz, 4H), 8.34 (*t*, 8.0 Hz, 4H), 8.26 (*t,* 8.0 Hz, 4H), 7.62 (*d*, 8.0 Hz, 8H), 7.13 (*d,* 8.0 Hz, 8H), 7.05 (*t,* 4.0 Hz, 4H).

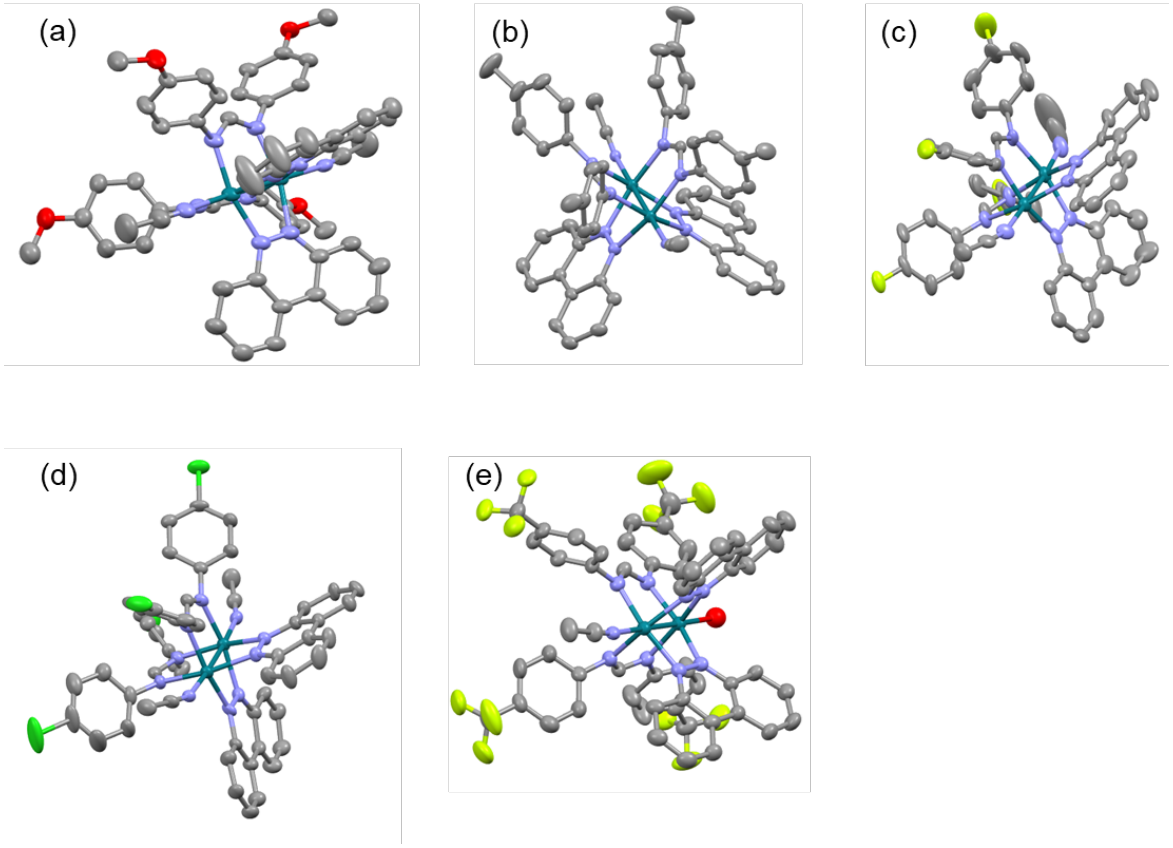

Figure S1. Structures of (a) **1**, (b) **2**, (c) **4**, (d) **5**, and (e) **6** obtained using single-crystal x-ray diffraction (hydrogen atoms and $[BF_4]^-$ counterions were omitted for clarity; ellipsoids are drawn at 50% probability).

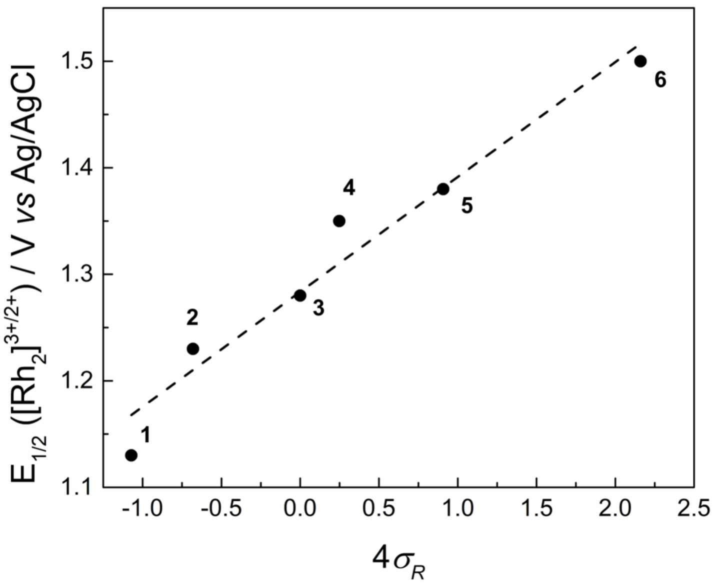

Figure S2. $E_{1/2}([Rh_2]^{3+/2+})$ *vs* $4\sigma_R$ values for **1** – **6** in CH_3CN (0.1 M $TBAPF_6$).

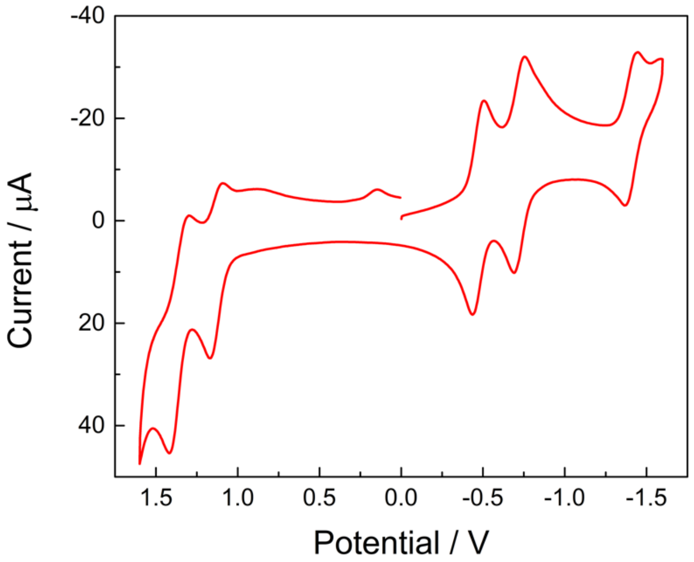

Figure S3. Cyclic voltammetry of **1** in CH_3CN (*vs* Ag/AgCl, 0.1 M $TBAPF_6$).

Table S1. Rh-Rh Bond Distance, $d_{Rh\text{-}Rh}$, Experimental and Calculated HOMO – LUMO Gaps, ΔE_{exp} and ΔE_{calc}, Respectively, and Calculated Energy of the First Singlet Excited State, $E^{1}{}_{ML\text{-}LCT}$, for **1** – **6** in CH_3CN.

Complex	$d_{Rh\text{-}Rh}$ / Å	ΔE_{exp}/ V[a]	ΔE_{calc} / eV	$E^{1}{}_{ML\text{-}LCT}$ / eV
**1**	2.3997(5)	1.60	2.20	1.64
**2**	2.4104(5)	1.69	2.26	1.67
**3**	2.4049[b]	1.73[b]	2.34	1.74
**4**	2.4004(6)	1.78	2.39	1.79
**5**	2.4078(7)	1.80	2.49	1.91
**6**	2.3866(6)	1.88	2.58	1.98

[a]From the difference in the first oxidation and first reduction potential. [b]From ref. S1.

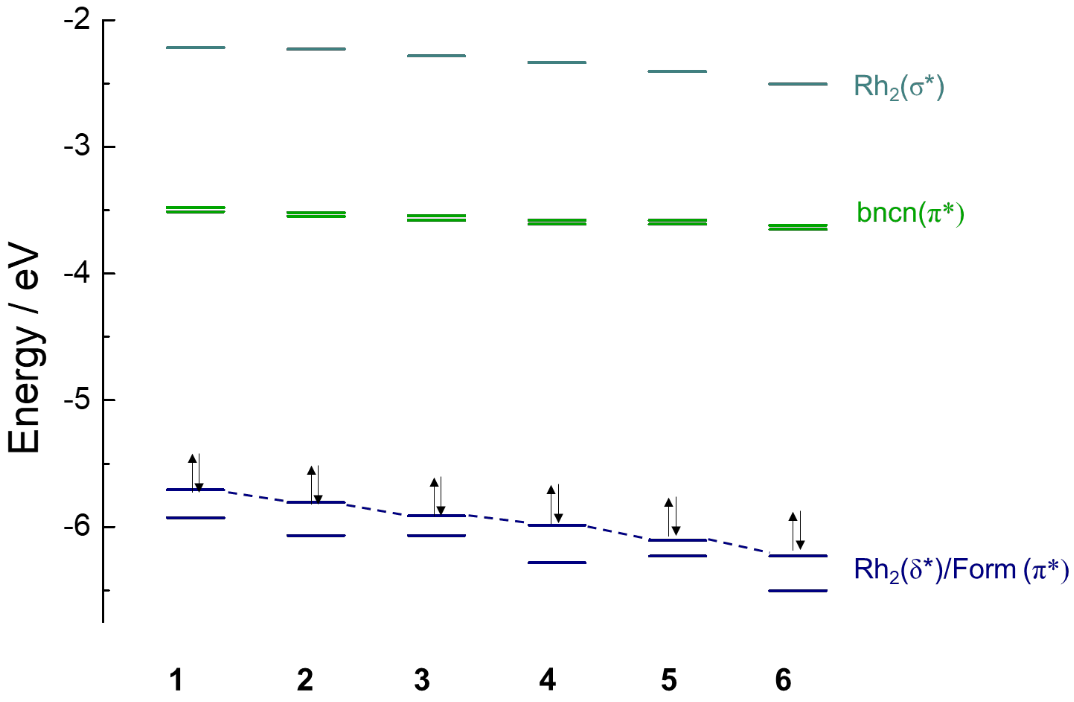

Figure S4. Calculated MO diagrams for **1 – 6** in CH_3CN.

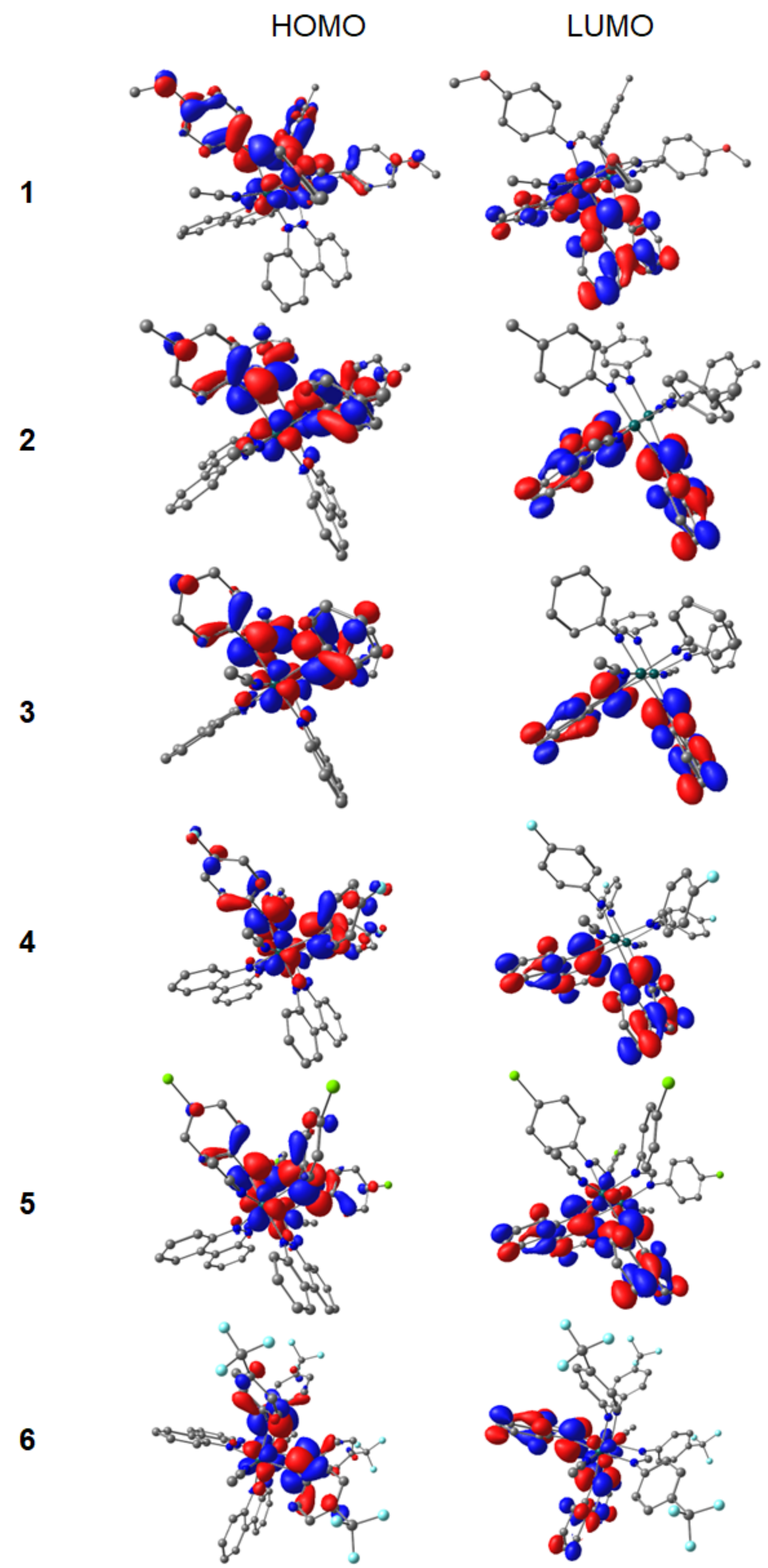

Figure S5. Electron density of the calculated HOMOs and LUMOs for **1 – 6** (plotted at 0.2 isovalue; hydrogen atoms are omitted for clarity).

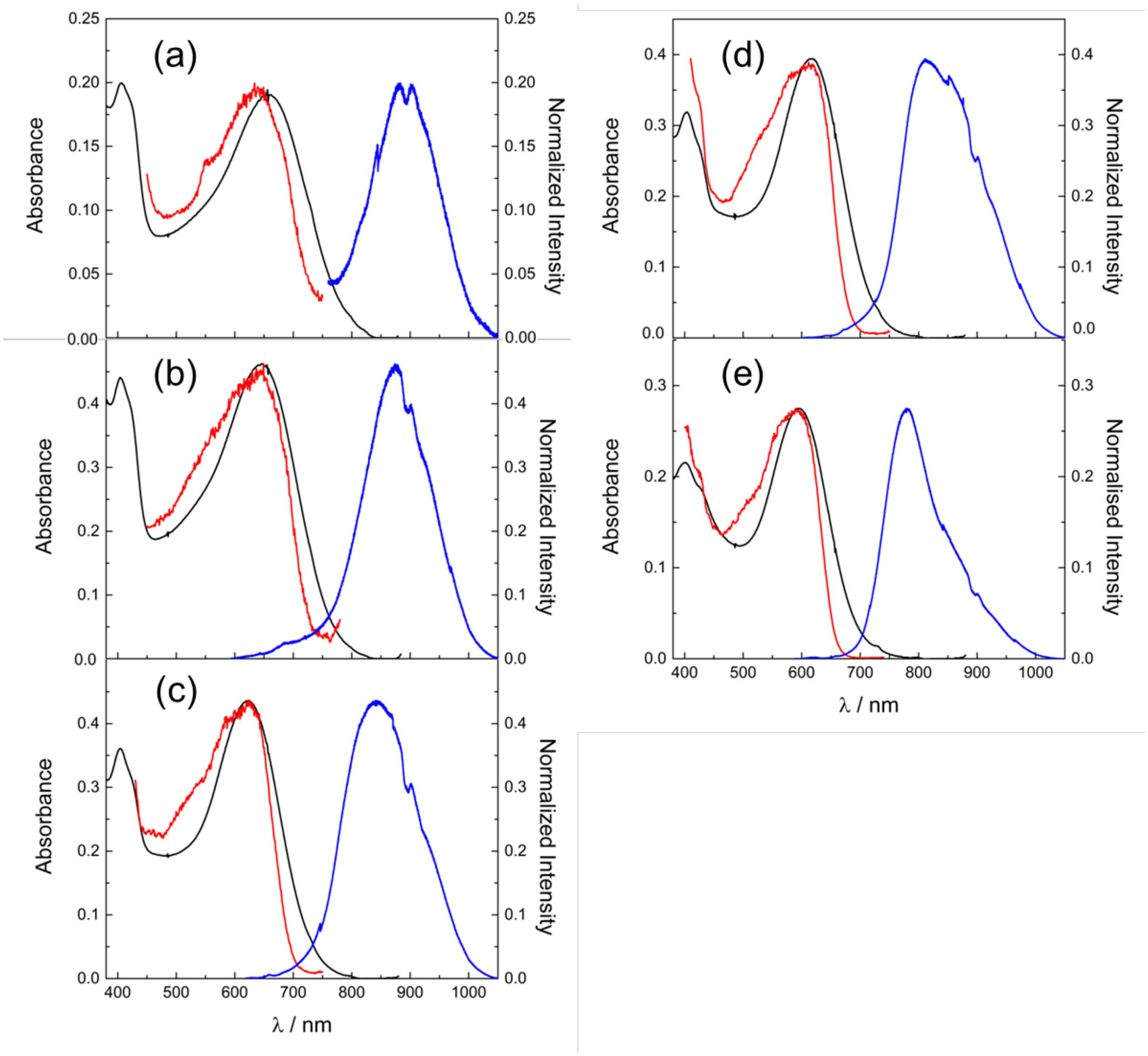

Figure S6. Emission (blue), excitation (red), and absorption (black) spectra of (a) **1** (λ_{exc} = 640 nm; λ_{em} = 820 nm) (b) **2** (λ_{exc} = 560 nm; λ_{em} = 840 nm), (c) **4** (λ_{exc} = 550 nm; λ_{em} = 800 nm), (d) **5** (λ_{exc} = 550 nm; λ_{em} = 770 nm), and (e) **6** (λ_{exc} = 550 nm; λ_{em} = 760 nm) in CH_3CN at 77 K.

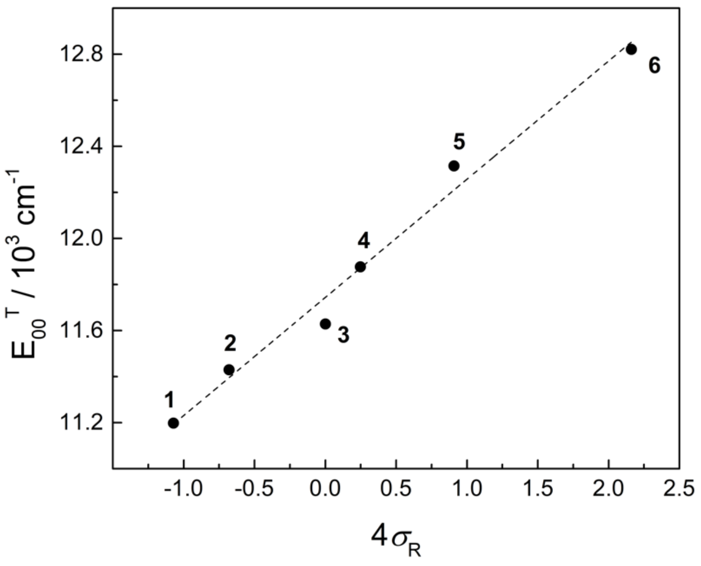

Figure S7. ^{3}ML-LCT energies, E_{00}^{T}, *vs* $4\sigma_R$ values for **1** – **6** in CH_3CN.

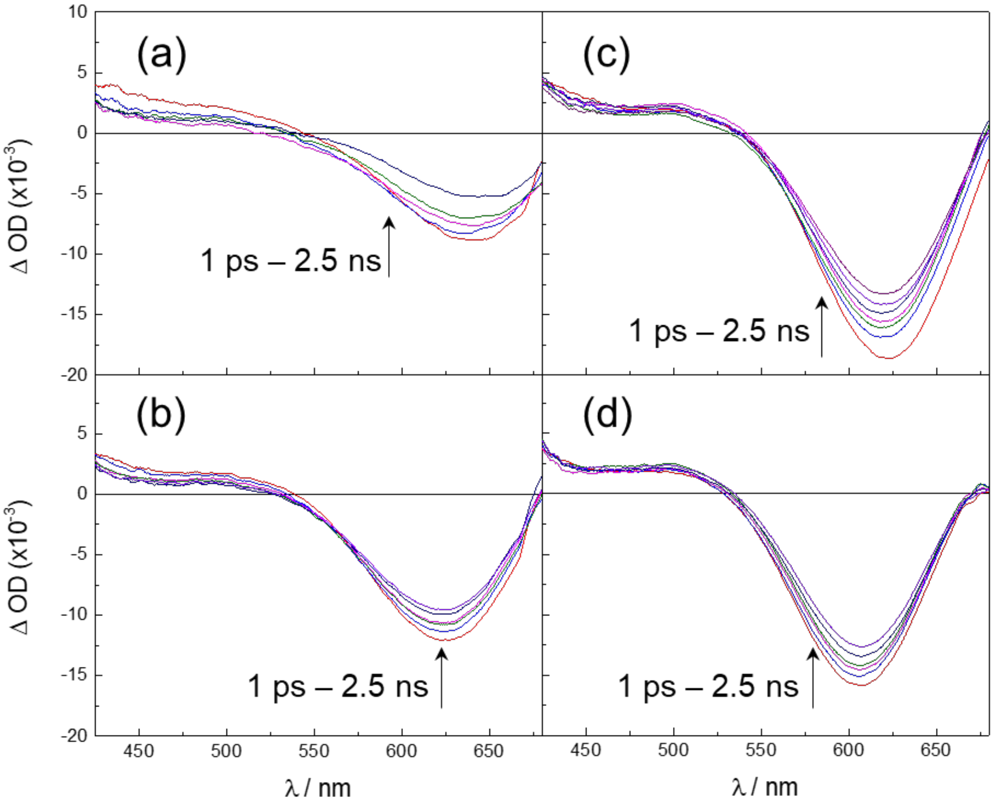

Figure S8. Transient absorption spectra of (a) **2**, (b) **4**, (c) **5**, and (d) **6** collected from 1 ps – 2.5 ns after the 400 nm (fwhm = 85 fs) excitation pulse in CH_3CN at room temperature.

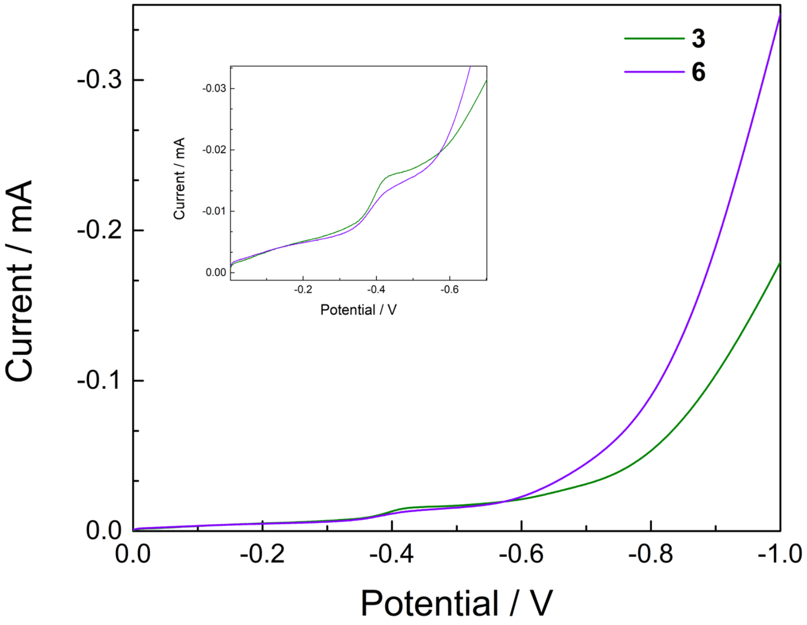

Figure S9. Catalytic currents observed **3** and **6** in the presence of 0.1 M TsOH scanned from 0 to −1.0 V *vs* Ag/AgCl in CH_3CN (scan rate = 200 mV/s). Inset: Expanded plot from 0.0 to −0.7 V.

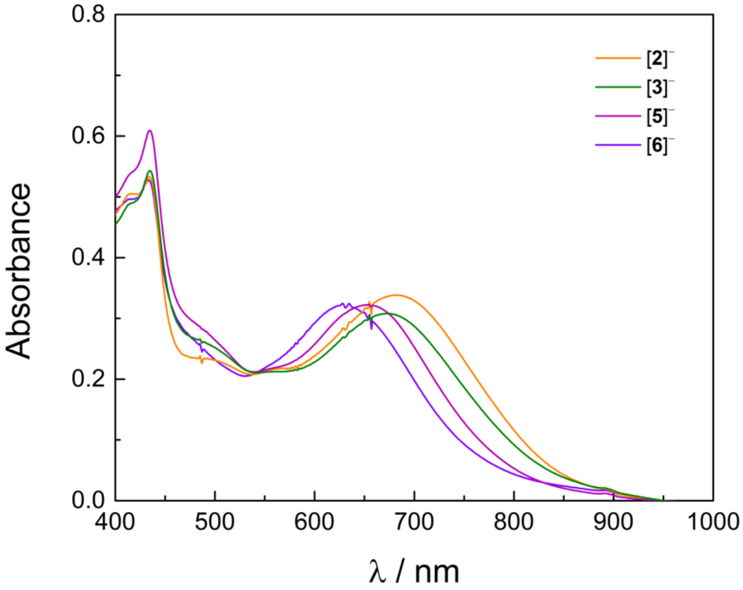

Figure S10. Steady state absorption spectra of [**2**]$^-$, [**3**]$^-$, [**5**]$^-$, and [**6**]$^-$ in CH_3CN.

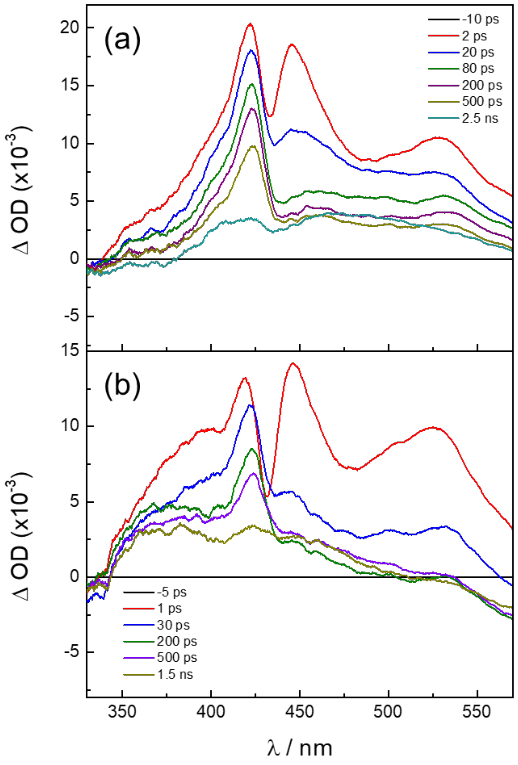

Figure S11. Transient absorption spectra of (a) [**3**]$^-$ and (b) [**5**]$^-$ collected from 1 ps – 2.5 ns after the 650 nm (fwhm = 85 fs) excitation pulse in CH_3CN at room temperature.

Table S2. Calculated Energies and Characters of the Frontier MOs and Doublet Excited State Lifetimes, τ_D, for [**1**]$^-$ – [**6**]$^-$ in CH_3CN.

		MO Energy / eV					
Character	MO	[**1**]$^-$	[**2**]$^-$	[**3**]$^-$	[**4**]$^-$	[**5**]$^-$	[**6**]$^-$
Rh_2-L(σ*)	LUMO+3	–1.48	–1.54	–1.60	–1.65	–1.71	–1.91
Rh_2(σ*)	LUMO+2	–1.75	–1.75	–1.80	–1.85	–1.94	–2.03
bncn(π*)	LUMO+1	–1.99	–1.98	–2.00	–2.03	–2.06	–2.08
bncn(π*)	LUMO	–3.27	–3.32	–3.35	–3.37	–3.36	–3.43
bncn$^-$ (π*)	HDOMO	–4.14	–4.19	–4.21	–4.24	–4.23	–4.30
Rh_2(δ*)/Form(π*)	SOMO-1	–5.44	–5.49	–5.59	–5.66	–5.80	–5.92
τ_D / ns		*a*	1.2	0.49	*b*	0.43	0.26

[a]Decomposed when exposed to laser. [b]Not measured.

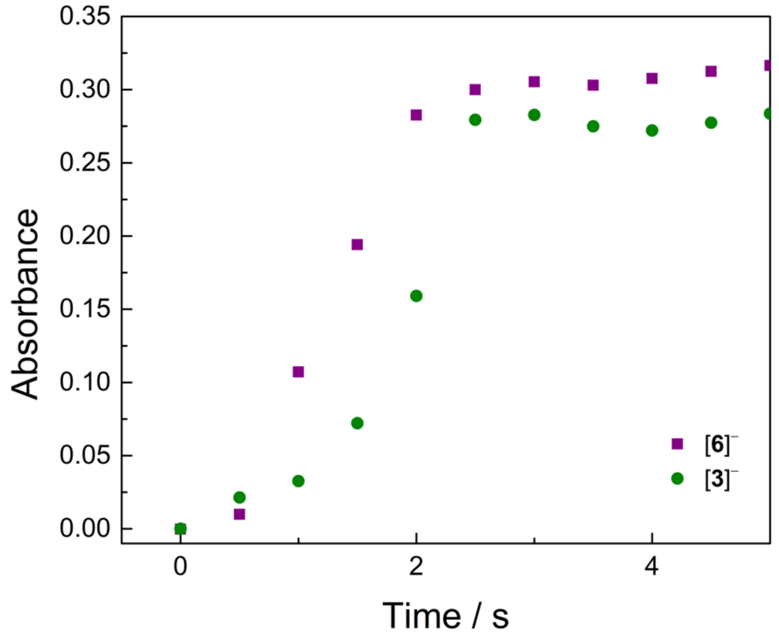

Figure S12. Changes in the absorption of [**3**]$^{-}$ at 633 nm (green circles) and [**6**]$^{-}$ at 595 nm (purple squares) as a function of time upon the addition of excess TsOH in DMF.

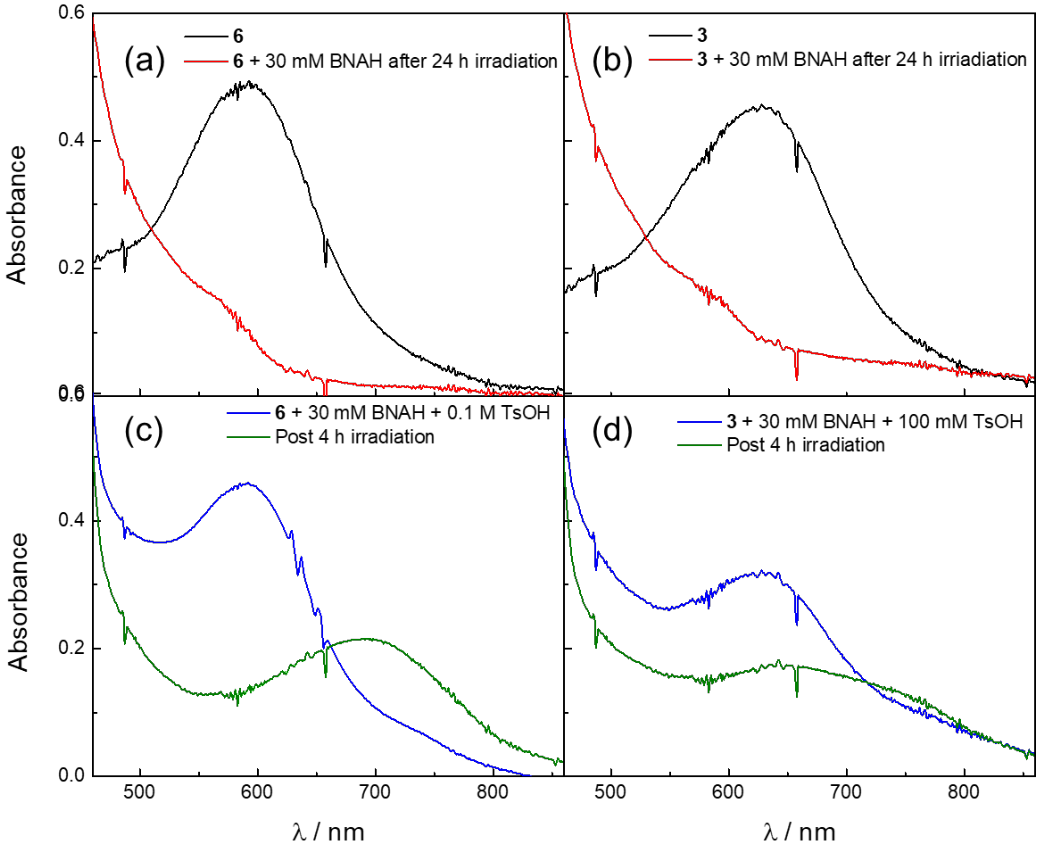

Figure S13. Spectral changes upon irradiation of solutions of (a) **6** and (b) **3** containing 30 mM BNAH and (c) **6** and (d) **3** containing 30 mM BNAH and 100 mM TsOH in DMF (λ_{irr} = 595 nm).

Single Crystal X-Ray Diffraction (XRD)

***Cis*-[Rh_2(*p*-OMe-Form)$_2$(bncn)$_2$]$^{2+}$ (1).** A 0.151 x 0.099 x 0.093 mm piece of a pruple block was mounted on a MiTeGen MicroMount with Paratone 24EX oil. Data were collected in a nitrogen gas stream at 100(2) K using ϕ and ϖ scans. Crystal-to-detector distance was 60 mm using variable exposure time (2s-10s) depending on q with a scan width of 0.75°. Data collection was 99.9% complete to 25.00° in *q* (0.83Å). A total of 90468 reflections were collected covering the indices, -29<=h<=32, -14<=k<=14, -26<=l<=26. 11854 reflections were found to be symmetry independent, with a R_{int} of 0.0429. Indexing and unit cell refinement indicated a *C*-centered, monoclinic lattice. The space group was found to be *C*c. The data were integrated using the Bruker SAINT software program and scaled using the SADABS software program. Solution by dual-space method (SHELXT) produced a complete phasing model for refinement. All nonhydrogen atoms were refined anisotropically by full-matrix least-squares (SHELXL-2014). All hydrogen atoms were placed using a riding model. Their positions were constrained relative to their parent atom using the appropriate HFIX command in SHELXL-2014. Crystallographic data are summarized in Table S3 and S4.

***Cis*-[Rh_2(*p*-CH_3-Form)$_2$(bncn)$_2$]$^{2+}$ (2).** A 0.275 x 0.180 x 0.122 mm piece of a red block was mounted on a MiTeGen MicroMount with Paratone 24EX oil. Data were collected in a nitrogen gas stream at 100(2) K using ϕ and ϖ scans. Crystal-to-detector distance was 60 mm using variable exposure time (3s-10s) depending on q with a scan width of 1.0°. Data collection was 99.8% complete to 25.00° in *q* (0.83Å). A total of 83434 reflections were collected covering the indices, -15<=h<=15, -26<=k<=26, -18<=l<=18. 7555 reflections were found to be symmetry independent, with a R_{int} of 0.0511. Indexing and unit cell refinement indicated a primitive, monoclinic lattice. The space group was found to be $P2_1$/m. The data were integrated using the Bruker SAINT software program and scaled using the SADABS software program. Solution by dual-space method (SHELXT) produced a complete phasing model for refinement. All nonhydrogen atoms were refined anisotropically by full-matrix least-squares (SHELXL-2014). All hydrogen atoms were placed using a riding model. Their positions were constrained relative to their parent atom using the appropriate HFIX command in SHELXL-2014. Crystallographic data are summarized in Table S5 and S6.

***Cis*-[Rh_2(*p*-F-Form)$_2$(bncn)$_2$]$^{2+}$ (4).** A 0.161 x 0.135 x 0.042 mm piece of a purple plate was mounted on a MiTeGen MicroMount with Paratone 24EX oil. Data were collected in a nitrogen gas stream at 100(2) K using ϕ and ϖ scans. Crystal-to-detector distance was 60 mm using variable exposure time (2s-5s) depending on θ with a scan width of 1.0°. Data collection was 99.9% complete to 25.00° in *θ* (0.83Å). A total of 152018 reflections were collected covering the indices, -24<=h<=24, -17<=k<=17, -22<=l<=22. 9957 reflections were found to be symmetry independent, with a R_{int} of 0.0747. Indexing and unit cell refinement indicated a primitive, monoclinic lattice. The space group was found to be $P2_1$/c. The data were integrated using the Bruker SAINT software program and scaled using the SADABS software program. Solution by dual-space method (SHELXT) produced a complete phasing model for refinement. All nonhydrogen atoms were refined anisotropically by full-matrix least-squares (SHELXL-2014). All hydrogen atoms were placed using a riding model. Their positions were constrained relative to

their parent atom using the appropriate HFIX command in SHELXL-2014. Crystallographic data are summarized in Table S7 and S8.

***Cis*-[Rh_2(*p*-Cl-Form)$_2$(bncn)$_2$]$^{2+}$ (5).** A 0.470 x 0.234 x 0.057 mm piece of a purple plank was mounted on a MiTeGen MicroMount with Paratone 24EX oil. Data were collected in a nitrogen gas stream at 100(2) K using ϕ and ϖ scans. Crystal-to-detector distance was 80 mm using variable exposure time (2s-30s) depending on θ with a scan width of 0.75°. Data collection was 99.9% complete to 25.00° in *θ* (0.83Å). A total of 690629 reflections were collected covering the indices, -34<=h<=34, -20<=k<=24, -51<=l<=51. 23099 reflections were found to be symmetry independent, with a R_{int} of 0.0809. Indexing and unit cell refinement indicated a primitive, orthorhombic lattice. The space group was found to be *P*bca. The data were integrated using the Bruker SAINT software program and scaled using the SADABS software program. Solution by dual-space method (SHELXT) produced a complete phasing model for refinement. All nonhydrogen atoms were refined anisotropically by full-matrix least-squares (SHELXL-2014). All hydrogen atoms were placed using a riding model. Their positions were constrained relative to their parent atom using the appropriate HFIX command in SHELXL-2014. Due to unmodelable solvent disorder, Olex2 solvent mask was used to remove the electron density from the lattice due to the disordered solvent contribution. Solvent appeared to be Diethyl ether. Four voids were found with approximately 249 electrons in each, which is consistent with 1.5 molecules of Diethyl ether per molecule of interest. Crystallographic data are summarized in Table S9 and S10.

***Cis*-[Rh_2(*p*-CF_3-Form)$_2$(bncn)$_2$]$^{2+}$ (6).** A 0.040 x 0.029 x 0.021 mm piece of a red block was mounted on a MiTeGen MicroMount with Paratone 24EX oil. Data were collected in a nitrogen gas stream at 100(2) K using ϕ and ϖ scans. Crystal-to-detector distance was 60 mm using variable exposure time (5s-60s) depending on q with a scan width of 1.0°. Data collection was 100% complete to 25.00° in *q* (0.83Å). A total of 105614 reflections were collected covering the indices, -14<=h<=14, -16<=k<=16, -23<=l<=23. 10963 reflections were found to be symmetry independent, with a R_{int} of 0.1083. Indexing and unit cell refinement indicated a primitive, triclinic lattice. The space group was found to be *P*-1. The data were integrated using the Bruker SAINT software program and scaled using the SADABS software program. Solution by dual-space method (SHELXT) produced a complete phasing model for refinement. All nonhydrogen atoms were refined anisotropically by full-matrix least-squares (SHELXL-2014). All hydrogen atoms were placed using a riding model. Their positions were constrained relative to their parent atom using the appropriate HFIX command in SHELXL-2014. Crystallographic data are summarized in Table S11 and S12.

Table S3. Crystal Data and Structure Refinement for **1**.

Report date	2025-12-11
Identification code	p-OMe-Form
Empirical formula	C63.09 H63.63 B2 F8 N10.54 O5 Rh2
Molecular formula	C58 H52 N10 O4 Rh2, C4 H10 O, 2(B F4), 0.544(C2 H3 N)
Formula weight	1428.96
Temperature	100.0 K
Wavelength	0.71073 Å
Crystal system	Monoclinic
Space group	Cc
Unit cell dimensions	a = 25.8563(13) Å α= 90°.
	b = 11.5969(6) Å β= 108.868(2)°.
	c = 21.5507(11) Å γ = 90°.
Volume	6114.8(5) $Å^3$
Z	4
Density (calculated)	1.552 Mg/m^3
Absorption coefficient	0.624 mm^{-1}
F(000)	2912
Crystal size	0.151 x 0.099 x 0.093 mm^3
Crystal color, habit	purple block
Theta range for data collection	2.058 to 26.416°.
Index ranges	-29<=h<=32, -14<=k<=14, -26<=l<=26
Reflections collected	90468
Independent reflections	11854 [R(int) = 0.0429, R(sigma) = 0.0245]
Completeness to theta = 25.000°	99.9 %
Absorption correction	Semi-empirical from equivalents
Max. and min. transmission	0.2602 and 0.2304
Refinement method	Full-matrix least-squares on F^2
Data / restraints / parameters	11854 / 475 / 1031
Goodness-of-fit on F^2	1.065
Final R indices [I>2sigma(I)]	R1 = 0.0299, wR2 = 0.0701
R indices (all data)	R1 = 0.0346, wR2 = 0.0737
Absolute structure parameter	-0.005(5)
Extinction coefficient	n/a
Largest diff. peak and hole	0.534 and -0.386 e.$Å^{-3}$

Table S4. Atomic Coordinates (x 10^4) and Equivalent Isotropic Displacement Parameters (Å^2 x 10^3) for **1**, where U(eq) is Defined as One third of the Trace of the Orthogonalized U^{ij} Tensor.

	x	y	z	U(eq)
Rh(1)	6094(1)	4429(1)	5368(1)	33(1)
Rh(2)	5283(1)	5186(1)	4577(1)	35(1)
O(1)	6583(2)	-784(3)	7041(2)	44(1)
O(2)	2578(2)	4154(4)	3976(2)	63(1)
O(3)	6748(2)	4215(4)	8764(2)	49(1)
O(4)	4283(2)	10617(3)	5035(2)	59(1)
N(1)	5653(2)	3019(3)	5477(2)	34(1)
N(2)	4850(2)	3863(3)	4794(2)	36(1)
N(3)	5869(2)	5223(3)	6088(2)	36(1)
N(4)	5144(2)	6159(3)	5300(2)	39(1)
N(5)	6110(2)	3788(4)	4487(2)	37(1)
N(6)	5635(2)	4060(4)	4064(2)	37(1)
N(7)	6318(2)	6005(3)	5071(2)	35(1)
N(8)	5884(2)	6362(3)	4607(2)	39(1)
N(9)	6832(2)	3712(3)	6091(2)	32(1)
N(10)	4522(2)	5844(4)	3817(2)	41(1)
C(1)	5119(2)	3039(4)	5201(2)	36(1)
C(2)	5463(2)	5957(4)	5909(2)	37(1)
C(3)	5889(2)	2034(4)	5867(2)	34(1)
C(4)	6262(2)	1351(4)	5702(3)	38(1)
C(5)	6506(2)	406(4)	6084(3)	38(1)
C(6)	6365(2)	132(4)	6633(2)	36(1)
C(7)	5989(2)	804(4)	6799(3)	41(1)
C(8)	5752(2)	1751(4)	6423(2)	37(1)
C(9)	6969(3)	-1482(5)	6878(3)	51(1)
C(10)	4276(2)	3859(4)	4587(3)	38(1)
C(11)	3967(3)	3406(6)	3988(3)	56(2)
C(12)	3400(3)	3484(6)	3770(3)	57(2)
C(13)	3138(3)	4012(5)	4162(3)	50(1)
C(14)	3436(2)	4440(4)	4764(3)	47(1)
C(15)	3994(2)	4360(4)	4974(3)	42(1)

C(16)	2255(3)	3802(8)	3340(4)	74(2)
C(17)	6094(2)	4996(4)	6777(2)	35(1)
C(18)	6627(2)	5308(4)	7113(2)	40(1)
C(19)	6861(2)	5070(4)	7784(3)	39(1)
C(20)	6550(2)	4502(4)	8110(2)	39(1)
C(21)	6010(2)	4222(4)	7776(3)	40(1)
C(22)	5786(2)	4466(4)	7114(2)	37(1)
C(23)	7322(3)	4357(6)	9078(3)	54(1)
C(24)	4901(2)	7284(4)	5205(2)	37(1)
C(25)	4351(2)	7445(5)	4935(3)	46(1)
C(26)	4124(2)	8550(5)	4865(3)	50(1)
C(27)	4461(2)	9481(4)	5074(3)	45(1)
C(28)	5017(2)	9340(4)	5336(3)	42(1)
C(29)	5236(2)	8252(4)	5403(3)	40(1)
C(30)	3710(3)	10816(6)	4820(3)	60(2)
C(31)	6490(2)	3161(4)	4307(2)	39(1)
C(32)	7007(3)	2981(5)	4762(3)	50(1)
C(33)	7390(3)	2349(5)	4602(3)	54(1)
C(34)	7263(3)	1871(5)	3970(3)	56(2)
C(35)	6762(3)	2035(4)	3514(3)	50(1)
C(36)	6354(2)	2687(4)	3668(2)	40(1)
C(37)	5822(2)	2887(4)	3227(2)	40(1)
C(38)	5474(2)	3611(4)	3437(2)	40(1)
C(39)	4946(3)	3843(6)	3027(3)	53(1)
C(40)	4756(3)	3347(6)	2415(3)	61(2)
C(41)	5101(3)	2615(6)	2212(3)	57(2)
C(42)	5615(3)	2393(5)	2594(3)	49(1)
C(43)	6786(2)	6650(4)	5288(2)	36(1)
C(44)	7244(2)	6200(4)	5780(3)	41(1)
C(45)	7709(2)	6817(5)	6002(3)	45(1)
C(46)	7744(3)	7915(5)	5752(3)	52(1)
C(47)	7300(2)	8378(5)	5273(3)	49(1)
C(48)	6815(2)	7756(4)	5026(3)	40(1)
C(55)	7141(3)	3300(5)	6517(3)	52(1)
C(56)	7532(4)	2742(8)	7073(4)	91(3)
C(57)	4106(3)	6055(6)	3477(3)	59(2)

C(58)	3574(4)	6332(10)	3039(5)	107(3)
C(49)	6363(8)	8062(13)	4484(11)	36(4)
C(50)	5903(10)	7332(13)	4282(13)	35(4)
C(51)	5458(6)	7654(11)	3757(7)	43(3)
C(52)	5444(6)	8647(11)	3438(7)	55(3)
C(53)	5882(6)	9403(10)	3640(7)	54(3)
C(54)	6340(7)	9144(11)	4159(7)	44(3)
C(49B)	6315(10)	8273(16)	4572(14)	36(4)
C(50B)	5859(12)	7530(17)	4361(17)	35(4)
C(51B)	5349(7)	7903(14)	3888(8)	43(3)
C(52B)	5322(7)	9006(13)	3662(8)	55(3)
C(53B)	5776(7)	9732(13)	3864(8)	54(3)
C(54B)	6270(8)	9402(13)	4318(9)	44(3)
O(1S)	3906(6)	357(11)	6357(8)	116(3)
C(1S)	4412(10)	695(18)	6727(14)	116(3)
C(2S)	4413(13)	2013(19)	6800(14)	127(5)
C(3S)	3840(9)	-872(18)	6416(16)	126(5)
C(4S)	3263(6)	-1171(16)	6019(9)	97(5)
O(1SB)	4533(8)	1181(16)	6857(11)	116(3)
C(1SB)	4303(17)	2230(20)	6594(15)	127(5)
C(2SB)	4515(11)	3150(20)	7142(13)	123(7)
C(3SB)	4524(10)	67(18)	6738(16)	116(3)
C(4SB)	3991(12)	-600(30)	6640(20)	126(5)
F(1)	7851(7)	5111(17)	7166(8)	91(4)
F(2)	8205(10)	5022(17)	8262(9)	92(2)
F(3)	7808(5)	6650(12)	7712(10)	96(4)
F(4)	8638(5)	6087(15)	7670(8)	79(4)
B(1)	8150(8)	5730(18)	7721(10)	84(3)
F(1B)	8059(7)	5329(16)	7301(6)	101(4)
F(2B)	8443(5)	4188(11)	8149(6)	92(2)
F(3B)	8192(4)	5910(13)	8364(6)	82(3)
F(4B)	8903(5)	5770(14)	7970(9)	102(4)
B(1B)	8413(7)	5324(16)	7937(9)	84(3)
F(1C)	7910(8)	6120(20)	7369(8)	97(5)
F(2C)	8232(12)	4760(16)	8152(11)	92(2)
F(3C)	8018(8)	6563(18)	8402(10)	97(5)

F(4C)	8759(7)	6363(18)	8084(11)	88(5)
B(1C)	8240(9)	5920(20)	8004(11)	84(3)
F(5)	8397(5)	2076(10)	6298(7)	110(4)
F(6)	7744(5)	771(15)	5891(7)	103(4)
F(7)	8242(7)	695(12)	6961(6)	105(4)
F(8)	8633(5)	250(13)	6226(7)	102(4)
B(2)	8247(7)	962(13)	6344(8)	89(3)
F(5B)	8532(11)	1511(18)	7001(13)	88(6)
F(6B)	7998(9)	820(20)	5998(10)	93(5)
F(7B)	7918(8)	122(17)	6931(10)	92(5)
F(8B)	8684(10)	-250(20)	6662(14)	108(6)
B(2B)	8281(9)	570(20)	6640(12)	89(3)
F(5C)	8675(7)	1317(17)	6072(8)	110(5)
F(6C)	7861(7)	510(20)	6127(12)	106(6)
F(7C)	8524(8)	1117(16)	7060(8)	72(4)
F(8C)	8694(9)	-401(17)	6458(10)	89(5)
B(2C)	8417(8)	664(19)	6432(11)	89(3)
N(1S)	5357(6)	1450(9)	4159(6)	72(4)
C(5S)	4916(8)	1108(13)	3864(8)	86(6)
C(6S)	4412(9)	680(20)	3507(11)	139(10)

Table S5. Crystal Data and Structure Refinement for **2**.

Report date	2025-12-09	
Identification code	DTolF	
Empirical formula	C65.87 H67.74 B2 Cl8 F8 N10 Rh2	
Molecular formula	C58 H52 N10 Rh2, 3.872(C2 H4 Cl2), 0.128(C H2 Cl2), 2(B F4)	
Formula weight	1662.52	
Temperature	100.00 K	
Wavelength	0.71073 Å	
Crystal system	Monoclinic	
Space group	P 1 21/m 1	
Unit cell dimensions	a = 12.3492(10) Å	α= 90°.
	b = 21.4357(14) Å	β= 114.177(2)°.
	c = 14.8173(11) Å	γ = 90°.
Volume	3578.3(5) $Å^3$	
Z	2	
Density (calculated)	1.543 Mg/m^3	
Absorption coefficient	0.829 mm^{-1}	
F(000)	1682	
Crystal size	0.275 x 0.180 x 0.122 mm^3	
Crystal color, habit	red block	
Theta range for data collection	2.945 to 26.434°.	
Index ranges	-15<=h<=15, -26<=k<=26, -18<=l<=18	
Reflections collected	83434	
Independent reflections	7555 [R(int) = 0.0511, R(sigma) = 0.0241]	
Completeness to theta = 25.000°	99.8 %	
Absorption correction	Semi-empirical from equivalents	
Max. and min. transmission	0.0932 and 0.0680	
Refinement method	Full-matrix least-squares on F^2	
Data / restraints / parameters	7555 / 71 / 520	
Goodness-of-fit on F^2	1.061	
Final R indices [I>2sigma(I)]	R1 = 0.0349, wR2 = 0.0711	
R indices (all data)	R1 = 0.0546, wR2 = 0.0827	
Extinction coefficient	n/a	
Largest diff. peak and hole	0.619 and -0.665 e.$Å^{-3}$	

Table S6. Atomic Coordinates (x 10^4) and Equivalent Isotropic Displacement Parameters (Å^2 x10^3) for **2**, where U(eq) is Defined as One third of the Trace of the Orthogonalized U^{ij} Tensor.

	x	y	z	U(eq)
Rh(1)	7087(1)	7500	7249(1)	22(1)
Rh(2)	7015(1)	7500	8851(1)	23(1)
N(1)	8388(2)	8166(1)	7752(2)	24(1)
N(2)	8305(2)	8169(1)	9305(2)	25(1)
N(3)	5848(2)	6833(1)	7163(2)	25(1)
N(4)	5820(2)	6830(1)	8041(2)	26(1)
N(5)	7203(3)	7500	5797(2)	20(1)
N(6)	6993(3)	7500	10340(2)	21(1)
C(1)	8753(3)	8354(1)	8680(2)	25(1)
C(2)	9038(3)	8422(1)	7230(2)	26(1)
C(3)	10267(3)	8357(1)	7614(2)	30(1)
C(4)	10912(3)	8644(2)	7152(2)	35(1)
C(5)	10352(3)	8993(2)	6295(2)	36(1)
C(6)	9122(3)	9038(2)	5901(2)	34(1)
C(7)	8469(3)	8763(1)	6367(2)	30(1)
C(8)	11067(3)	9322(2)	5823(3)	52(1)
C(9)	8836(3)	8437(1)	10272(2)	27(1)
C(10)	8169(3)	8806(2)	10623(2)	36(1)
C(11)	8677(4)	9071(2)	11553(3)	45(1)
C(12)	9863(4)	8981(2)	12158(3)	50(1)
C(13)	10525(3)	8612(2)	11809(3)	50(1)
C(14)	10019(3)	8338(2)	10874(2)	37(1)
C(15)	10406(5)	9275(3)	13173(3)	86(2)
C(16)	5192(2)	6412(1)	6445(2)	25(1)
C(17)	5183(3)	6471(2)	5491(2)	30(1)
C(18)	4581(3)	6044(2)	4778(2)	34(1)
C(19)	3984(3)	5548(2)	4991(2)	35(1)
C(20)	3971(3)	5486(2)	5909(2)	32(1)
C(21)	4563(2)	5926(1)	6654(2)	26(1)
C(22)	4561(3)	5913(1)	7622(2)	27(1)
C(23)	3982(3)	5454(2)	7944(2)	32(1)

C(24)	3976(3)	5492(2)	8864(2)	37(1)
C(25)	4524(3)	5991(2)	9498(2)	38(1)
C(26)	5108(3)	6439(2)	9213(2)	36(1)
C(27)	5151(3)	6397(1)	8280(2)	27(1)
C(28)	7717(4)	7500	5318(3)	30(1)
C(29)	8435(5)	7500	4750(4)	46(1)
C(30)	7392(4)	7500	11176(3)	31(1)
C(31)	7931(5)	7500	12252(3)	50(1)
Cl(1S)	8456(1)	10648(1)	10400(1)	67(1)
Cl(2S)	7335(1)	9775(1)	7568(1)	58(1)
C(1S)	8459(3)	10200(2)	9389(3)	49(1)
C(2S)	7284(4)	10239(2)	8557(3)	61(1)
Cl(3S)	1060(2)	7500	3845(2)	108(1)
Cl(4S)	3151(1)	7500	6950(1)	57(1)
C(3S)	2354(7)	7500	4994(6)	88(3)
C(4S)	1922(6)	7500	5795(5)	57(2)
Cl(5S)	12586(4)	7405(6)	10107(3)	100(3)
Cl(6S)	13017(4)	7630(6)	12500(4)	110(3)
C(5S)	13859(9)	7287(6)	11115(8)	88(4)
C(6S)	14088(9)	7632(9)	12035(9)	82(4)
Cl(3B)	1280(20)	7774(15)	4338(16)	108(1)
Cl(4B)	2825(12)	7403(9)	6232(11)	57(1)
C(3SB)	2090(60)	7100(20)	5000(20)	88(3)
Cl(5B)	12980(30)	7500	9810(20)	100(3)
Cl(6B)	12870(40)	7500	12170(30)	110(3)
C(5SB)	14180(30)	7500	10970(20)	88(4)
C(6SB)	14140(40)	7500	11950(30)	82(4)
F(4)	7228(2)	9188(1)	13297(2)	57(1)
B(1)	6227(4)	8814(2)	12873(3)	40(1)
F(1)	6115(3)	8627(2)	11943(3)	71(1)
F(2)	5223(3)	9155(2)	12769(3)	49(1)
F(3)	6325(4)	8304(2)	13457(3)	74(1)
F(1B)	6450(20)	8377(12)	12370(20)	40(5)
F(2B)	5240(30)	9130(14)	12370(20)	40(5)
F(3B)	6240(30)	8517(15)	13780(20)	44(6)

Table S7. Crystal Sata and Structure Refinement for **4**.

Report date	2025-12-10
Identification code	p-F-Form
Empirical formula	C56 H46 B2 F12 N10 O Rh2
Molecular formula	C54 H40 F4 N10 Rh2, 2(B F4), 0.5(C4 H10 O), 0.5(H2 O)
Formula weight	1330.47
Temperature	100.0 K
Wavelength	0.71073 Å
Crystal system	Monoclinic
Space group	P 1 21/c 1
Unit cell dimensions	a = 20.387(4) Å α= 90°.
	b = 14.404(2) Å β= 92.196(6)°.
	c = 18.497(3) Å γ = 90°.
Volume	5427.6(16) Å^3
Z	4
Density (calculated)	1.628 Mg/m^3
Absorption coefficient	0.701 mm^{-1}
F(000)	2672
Crystal size	0.161 x 0.135 x 0.042 mm^3
Crystal color, habit	purple plate
Theta range for data collection	2.032 to 25.378°.
Index ranges	-24<=h<=24, -17<=k<=17, -22<=l<=22
Reflections collected	152018
Independent reflections	9957 [R(int) = 0.0747, R(sigma) = 0.0284]
Completeness to theta = 25.000°	99.9 %
Absorption correction	Semi-empirical from equivalents
Max. and min. transmission	0.0916 and 0.0680
Refinement method	Full-matrix least-squares on F^2
Data / restraints / parameters	9957 / 424 / 1045
Goodness-of-fit on F^2	1.125
Final R indices [I>2sigma(I)]	R1 = 0.0454, wR2 = 0.0961
R indices (all data)	R1 = 0.0637, wR2 = 0.1059
Extinction coefficient	n/a
Largest diff. peak and hole	0.616 and -0.851 e.Å^{-3}

Table S8. Atomic Coordinates (x 10^4) and Equivalent Isotropic Displacement Parameters (Å^2 x 10^3) for **4**, where U(eq) is Defined as One Third of the Trace of the Orthogonalized Uij Tensor.

	x	y	z	U(eq)
Rh(1)	2199(1)	4151(1)	4320(1)	36(1)
Rh(2)	2712(1)	5624(1)	4123(1)	40(1)
F(1)	-610(1)	3140(2)	6179(2)	63(1)
F(3)	2389(1)	1118(2)	7127(1)	52(1)
F(2)	2169(7)	9774(7)	6021(5)	63(4)
F(4)	5682(5)	7287(11)	5562(6)	71(3)
F(2B)	2490(30)	9663(11)	6004(12)	129(10)
F(4B)	5863(9)	6928(15)	5334(12)	55(5)
N(1)	1540(2)	4805(2)	4961(2)	41(1)
N(2)	2131(2)	6182(3)	4882(2)	45(1)
N(3)	2808(2)	3983(3)	5211(2)	44(1)
N(4)	3402(2)	5302(3)	4919(2)	50(1)
N(5)	1709(2)	4716(3)	3426(2)	37(1)
N(6)	1936(2)	5560(3)	3378(2)	37(1)
N(7)	2951(2)	3895(3)	3633(2)	40(1)
N(8)	3164(2)	4720(3)	3460(2)	40(1)
N(9)	1736(2)	2801(3)	4539(2)	38(1)
N(10)	3198(2)	6985(3)	4007(2)	53(1)
C(1)	1661(2)	5679(3)	5137(2)	42(1)
C(2)	3291(2)	4574(4)	5327(3)	53(1)
C(3)	981(2)	4387(3)	5280(2)	39(1)
C(4)	520(2)	3907(3)	4849(2)	40(1)
C(5)	-14(2)	3482(3)	5154(3)	43(1)
C(6)	-88(2)	3566(3)	5885(3)	47(1)
C(7)	346(2)	4055(3)	6324(3)	49(1)
C(8)	884(2)	4465(3)	6021(2)	42(1)
C(15)	2731(2)	3257(3)	5732(3)	44(1)
C(16)	2365(2)	3416(4)	6332(3)	53(1)
C(17)	2244(2)	2688(4)	6809(3)	52(1)
C(18)	2506(2)	1839(3)	6666(2)	45(1)
C(19)	2891(3)	1670(4)	6089(3)	54(1)

C(20)	2998(3)	2396(4)	5612(3)	54(1)
C(27)	1214(2)	4389(3)	2958(2)	39(1)
C(28)	1036(2)	3453(3)	3000(2)	45(1)
C(29)	547(2)	3122(4)	2541(3)	53(1)
C(30)	224(2)	3720(4)	2042(3)	59(2)
C(31)	407(2)	4627(4)	1988(3)	52(1)
C(32)	914(2)	4995(4)	2438(2)	44(1)
C(33)	1139(2)	5933(4)	2415(2)	45(1)
C(34)	1669(2)	6192(3)	2884(2)	42(1)
C(35)	1923(2)	7100(4)	2888(3)	50(1)
C(36)	1637(3)	7763(4)	2450(3)	64(2)
C(37)	1095(3)	7518(5)	1991(3)	71(2)
C(38)	859(2)	6639(4)	1964(3)	58(2)
C(39)	3222(2)	3095(3)	3333(2)	41(1)
C(40)	2959(2)	2233(3)	3525(2)	45(1)
C(41)	3215(3)	1440(4)	3241(3)	54(1)
C(42)	3743(3)	1491(4)	2779(3)	59(1)
C(43)	4002(3)	2325(4)	2590(3)	56(1)
C(44)	3736(2)	3166(4)	2853(3)	47(1)
C(45)	3945(2)	4071(4)	2639(3)	53(1)
C(46)	3626(2)	4843(4)	2934(3)	46(1)
C(47)	3761(2)	5753(4)	2710(3)	51(1)
C(48)	4231(3)	5899(5)	2213(3)	67(2)
C(49)	4572(3)	5139(5)	1937(4)	80(2)
C(50)	4437(3)	4243(5)	2139(3)	70(2)
C(51)	1501(2)	2205(3)	4828(3)	43(1)
C(52)	1183(3)	1468(4)	5220(3)	58(1)
C(9)	2130(14)	7109(12)	5194(15)	38(4)
C(10)	2489(10)	7320(11)	5835(9)	37(3)
C(11)	2498(7)	8215(10)	6111(7)	38(3)
C(12)	2159(8)	8897(9)	5739(7)	44(3)
C(13)	1816(11)	8724(10)	5094(10)	57(4)
C(14)	1803(12)	7824(10)	4823(12)	49(5)
C(21)	3965(6)	5874(11)	5107(6)	40(2)
C(22)	4112(7)	6200(11)	5795(7)	46(2)
C(23)	4695(6)	6648(9)	5964(7)	49(2)

C(24)	5114(6)	6816(11)	5410(7)	52(3)
C(25)	4986(5)	6525(12)	4719(6)	51(3)
C(26)	4410(6)	6035(12)	4571(6)	48(3)
C(53)	3411(6)	7665(8)	4270(6)	51(3)
C(54)	3722(6)	8502(7)	4590(6)	76(3)
C(9B)	2280(20)	7110(20)	5130(20)	49(8)
C(10B)	2600(20)	7186(19)	5781(18)	65(9)
C(11B)	2710(30)	8053(17)	6080(15)	87(9)
C(12B)	2440(30)	8784(16)	5722(15)	86(9)
C(13B)	2070(20)	8745(16)	5070(20)	81(9)
C(14B)	2010(20)	7863(16)	4770(20)	61(8)
C(21B)	4093(12)	5640(20)	4984(15)	43(4)
C(22B)	4252(13)	6020(20)	5669(15)	45(5)
C(23B)	4853(13)	6491(19)	5770(16)	56(5)
C(24B)	5264(11)	6509(16)	5219(13)	41(4)
C(25B)	5113(10)	6138(18)	4553(13)	42(4)
C(26B)	4529(10)	5688(18)	4436(13)	42(4)
C(53C)	3100(30)	7760(40)	4090(30)	51(3)
C(54C)	3160(30)	8830(40)	4260(30)	76(3)
C(53B)	3654(13)	7425(18)	4052(13)	51(3)
C(54B)	4121(13)	8148(16)	4253(13)	76(3)
F(5)	997(3)	9768(4)	3804(4)	75(2)
F(6)	783(3)	11228(4)	3419(4)	81(2)
F(7)	1286(3)	10226(5)	2703(3)	94(2)
F(8)	1818(4)	10794(6)	3722(4)	78(2)
F(5B)	1152(9)	9579(11)	3439(12)	73(4)
F(6B)	706(9)	11011(13)	3726(10)	63(3)
F(7B)	1475(9)	10816(16)	2803(9)	86(4)
F(8B)	1822(11)	10645(16)	3989(11)	58(4)
B(1)	1240(3)	10511(5)	3418(4)	63(1)
F(9)	3395(3)	7960(5)	2374(4)	51(2)
F(10)	2947(3)	8913(4)	3184(3)	55(1)
F(11)	3087(4)	9418(4)	2033(3)	70(2)
F(12)	3949(3)	9242(4)	2806(4)	72(2)
B(2)	3362(6)	8861(8)	2593(6)	58(1)
F(9B)	3630(8)	7955(11)	2214(9)	70(4)

F(10B)	3316(8)	8917(12)	3092(9)	88(4)
F(11B)	4138(7)	9260(9)	2380(7)	71(3)
F(12B)	4313(5)	8100(8)	3195(6)	60(2)
B(2B)	3875(9)	8511(13)	2744(10)	58(1)
F(9C)	3288(10)	8099(16)	2716(12)	67(4)
F(10C)	4306(9)	8139(12)	2190(9)	65(4)
F(11C)	3614(11)	9434(12)	2186(10)	65(4)
F(12C)	4091(10)	8980(16)	3188(10)	72(4)
B(2C)	3848(11)	8636(16)	2526(12)	58(1)
O(1S)	4970(20)	9870(30)	4970(40)	169(11)
C(1S)	5606(15)	9900(20)	4673(19)	273(18)
C(2S)	5915(16)	9082(19)	5010(20)	214(11)
C(3S)	4597(13)	10616(14)	4676(13)	179(8)
C(4S)	3862(14)	10480(20)	4790(20)	214(11)
O(2S)	2347(9)	9616(9)	2041(7)	107(5)
O(3S)	440(20)	9150(30)	5022(19)	134(18)

Table S9. Crystal Data and Structure Refinement for **5**.

Report date	2025-12-10	
Identification code	p-Cl-Form	
Empirical formula	C60 H55 B2 Cl4 F8 N10 O1.50 Rh2	
Molecular formula	C54 H40 Cl4 N10 Rh2, 2(B F4), 1.5[C4H10O]	
Formula weight	1461.38	
Temperature	100.0 K	
Wavelength	0.71073 Å	
Crystal system	Orthorhombic	
Space group	Pbca	
Unit cell dimensions	a = 28.632(6) Å	α= 90°.
	b = 20.584(4) Å	β= 90°.
	c = 42.550(9) Å	γ = 90°.
Volume	25078(9) $Å^3$	
Z	16	
Density (calculated)	1.548 Mg/m^3	
Absorption coefficient	0.771 mm^{-1}	
F(000)	11792	
Crystal size	0.470 x 0.234 x 0.057 mm^3	
Crystal color, habit	purple plank	
Theta range for data collection	1.980 to 25.440°.	
Index ranges	-34<=h<=34, -20<=k<=24, -51<=l<=51	
Reflections collected	690629	
Independent reflections	23099 [R(int) = 0.0809, R(sigma) = 0.0276]	
Completeness to theta = 25.000°	99.9 %	
Absorption correction	Semi-empirical from equivalents	
Max. and min. transmission	0.0916 and 0.0662	
Refinement method	Full-matrix least-squares on F^2	
Data / restraints / parameters	23099 / 927 / 1720	
Goodness-of-fit on F^2	1.173	
Final R indices [I>2sigma(I)]	R1 = 0.0681, wR2 = 0.1407	
R indices (all data)	R1 = 0.0872, wR2 = 0.1528	
Extinction coefficient	n/a	
Largest diff. peak and hole	1.107 and -0.902 e.$Å^{-3}$	

Table S10. Atomic Coordinates (x 10^4) and Equivalent Isotropic Displacement Parameters (Å^2 x 10^3) for **5**, where U(eq) is Defined as One Third of the Trace of the Orthogonalized U^{ij} Tensor.

	x	y	z	U(eq)
Rh(1)	6722(1)	6865(1)	3566(1)	29(1)
Rh(2)	5958(1)	6469(1)	3705(1)	29(1)
Cl(1)	8978(1)	6346(1)	4347(1)	57(1)
Cl(2)	5204(1)	4074(1)	4862(1)	72(1)
Cl(3)	8069(1)	9693(1)	3893(1)	63(1)
Cl(4)	4393(1)	7763(2)	4932(1)	117(1)
N(1)	6981(2)	6399(2)	3958(1)	32(1)
N(2)	6245(2)	5982(2)	4077(1)	31(1)
N(3)	6605(2)	7675(2)	3837(1)	33(1)
N(4)	5881(2)	7268(2)	3995(1)	32(1)
N(5)	6648(2)	5991(2)	3332(1)	31(1)
N(6)	6226(2)	5784(2)	3399(1)	31(1)
N(7)	6273(2)	7252(2)	3238(1)	32(1)
N(8)	5857(2)	7041(2)	3312(1)	34(1)
N(9)	7432(2)	7248(2)	3462(1)	32(1)
N(10)	5271(2)	6127(2)	3855(1)	29(1)
C(1)	6691(2)	6064(3)	4136(2)	34(1)
C(2)	6220(2)	7701(3)	4003(1)	34(1)
C(3)	7461(2)	6382(3)	4053(1)	32(1)
C(4)	7801(2)	6107(3)	3860(2)	42(2)
C(5)	8264(2)	6093(4)	3948(2)	46(2)
C(6)	8387(2)	6337(3)	4236(2)	42(2)
C(7)	8059(2)	6595(3)	4437(2)	40(2)
C(8)	7595(2)	6622(3)	4344(2)	32(1)
C(9)	6004(2)	5527(3)	4275(1)	32(1)
C(10)	5980(2)	5610(3)	4599(2)	40(2)
C(11)	5738(2)	5153(4)	4781(2)	51(2)
C(12)	5524(2)	4633(4)	4639(2)	46(2)
C(13)	5556(2)	4535(3)	4322(2)	45(2)
C(14)	5801(2)	4979(3)	4140(2)	43(2)
C(15)	6930(2)	8194(3)	3863(2)	33(1)

C(16)	7220(2)	8247(3)	4124(2)	40(2)
C(17)	7562(2)	8715(3)	4133(2)	43(2)
C(18)	7610(2)	9136(3)	3887(2)	42(2)
C(19)	7318(3)	9114(4)	3632(2)	56(2)
C(20)	6972(3)	8640(3)	3622(2)	46(2)
C(21)	5511(2)	7377(3)	4216(2)	42(2)
C(22)	5622(3)	7300(4)	4539(2)	52(2)
C(23)	5277(3)	7416(4)	4761(2)	66(2)
C(24)	4841(3)	7607(5)	4657(2)	73(3)
C(25)	4740(3)	7682(6)	4354(2)	88(4)
C(26)	5085(2)	7566(5)	4127(2)	66(2)
C(27)	6940(2)	5644(3)	3136(1)	34(1)
C(28)	7374(2)	5921(3)	3056(1)	36(1)
C(29)	7677(2)	5581(3)	2872(2)	42(2)
C(30)	7552(2)	4960(3)	2760(2)	47(2)
C(31)	7132(2)	4688(3)	2833(2)	43(2)
C(32)	6812(2)	5024(3)	3022(2)	35(1)
C(33)	6353(2)	4795(3)	3098(2)	39(2)
C(34)	6057(2)	5207(3)	3274(1)	34(1)
C(35)	5592(2)	5045(3)	3334(2)	43(2)
C(36)	5423(3)	4463(4)	3225(2)	62(2)
C(37)	5717(3)	4024(4)	3063(2)	62(2)
C(38)	6174(3)	4192(3)	2997(2)	48(2)
C(39)	6346(2)	7671(3)	2987(2)	34(1)
C(40)	6801(2)	7864(3)	2913(2)	40(2)
C(41)	6869(3)	8308(3)	2675(2)	46(2)
C(42)	6486(3)	8563(4)	2507(2)	50(2)
C(43)	6043(3)	8362(4)	2573(2)	53(2)
C(44)	5957(2)	7904(3)	2813(2)	39(2)
C(45)	5505(2)	7642(3)	2883(2)	41(2)
C(46)	5466(2)	7206(3)	3136(1)	35(1)
C(47)	5037(2)	6918(4)	3211(2)	46(2)
C(48)	4655(2)	7047(4)	3031(2)	47(2)
C(49)	4687(2)	7483(4)	2781(2)	49(2)
C(50)	5096(2)	7787(4)	2710(2)	50(2)
C(51)	7794(2)	7465(3)	3494(2)	41(2)

C(52)	8259(2)	7741(4)	3544(2)	56(2)
C(53)	4981(2)	5996(3)	4024(2)	40(2)
C(54)	4622(3)	5839(4)	4256(2)	58(2)
Rh(1')	7379(1)	5372(1)	830(1)	27(1)
Rh(2')	7189(1)	5810(1)	1339(1)	30(1)
Cl(1')	8656(1)	6823(2)	-482(1)	43(1)
Cl(2')	7377(1)	9102(1)	1970(1)	42(1)
Cl(3')	6121(1)	5571(1)	-666(1)	68(1)
Cl(4')	5377(1)	8077(1)	1819(1)	83(1)
N(1')	7667(2)	6245(2)	716(1)	25(1)
N(2')	7477(2)	6680(3)	1207(1)	33(1)
N(3')	6751(2)	5700(3)	665(1)	29(1)
N(4')	6572(2)	6132(3)	1166(1)	34(1)
N(5')	7944(2)	5152(3)	1109(1)	32(1)
N(6')	7848(2)	5399(3)	1384(1)	37(1)
N(7')	7038(2)	4643(3)	1058(1)	32(1)
N(8')	6923(2)	4873(3)	1335(1)	36(1)
N(9')	7556(2)	5030(3)	352(1)	36(1)
N(10')	7032(2)	6187(3)	1811(1)	39(1)
C(1')	7644(2)	6731(3)	919(2)	31(2)
C(2')	6478(2)	6035(3)	861(2)	32(1)
C(3')	7905(2)	6381(3)	427(2)	27(1)
C(4')	8347(2)	6133(5)	370(2)	35(2)
C(5')	8579(3)	6271(6)	91(2)	35(1)
C(6')	8359(4)	6650(8)	-134(3)	34(1)
C(7')	7922(5)	6900(14)	-81(4)	35(2)
C(8')	7696(4)	6769(11)	199(3)	32(2)
C(9')	7465(2)	7272(3)	1383(2)	31(1)
C(10')	7233(3)	7811(3)	1272(2)	37(2)
C(11')	7210(3)	8377(3)	1450(2)	38(2)
C(12')	7421(3)	8402(4)	1736(3)	35(2)
C(13')	7676(3)	7878(3)	1847(2)	37(2)
C(14')	7700(2)	7312(3)	1668(2)	35(1)
C(15')	6596(2)	5668(3)	346(2)	31(1)
C(16')	6538(3)	6239(3)	173(2)	36(2)
C(17')	6382(3)	6213(4)	-138(2)	45(2)

C(18')	6294(3)	5618(4)	-274(2)	45(2)
C(19')	6354(3)	5044(4)	-105(2)	44(2)
C(20')	6500(3)	5075(4)	204(2)	38(2)
C(21')	6268(3)	6584(4)	1317(2)	40(2)
C(22')	6191(3)	7193(4)	1188(2)	49(2)
C(23')	5909(3)	7648(4)	1337(2)	61(2)
C(24')	5710(3)	7489(4)	1622(2)	58(2)
C(25')	5776(3)	6892(4)	1755(2)	55(2)
C(26')	6052(3)	6436(4)	1596(2)	49(2)
C(27')	8360(2)	4818(3)	1049(2)	36(1)
C(28')	8431(3)	4543(4)	745(2)	44(2)
C(29')	8837(3)	4234(4)	681(2)	48(2)
C(30')	9184(3)	4175(4)	909(2)	51(2)
C(31')	9118(3)	4418(4)	1202(2)	46(2)
C(32')	8696(3)	4746(3)	1286(2)	40(1)
C(33')	8598(3)	5000(4)	1585(2)	40(1)
C(34')	8172(3)	5353(4)	1630(2)	37(1)
C(35')	8072(3)	5653(4)	1912(2)	46(2)
C(36')	8395(3)	5615(4)	2156(2)	51(2)
C(37')	8803(3)	5254(4)	2124(2)	54(2)
C(38')	8902(3)	4948(4)	1850(2)	50(2)
C(39')	6894(2)	4028(3)	960(2)	36(2)
C(40')	7042(3)	3807(4)	666(2)	42(2)
C(41')	6867(3)	3228(4)	553(2)	49(2)
C(42')	6550(4)	2872(4)	729(3)	60(2)
C(43')	6421(4)	3068(4)	1027(3)	58(2)
C(44')	6595(3)	3654(3)	1148(2)	48(2)
C(45')	6481(3)	3884(3)	1457(2)	48(2)
C(46')	6651(2)	4515(3)	1542(2)	39(2)
C(47')	6542(3)	4789(4)	1835(2)	53(3)
C(48')	6273(4)	4439(4)	2044(3)	74(3)
C(49')	6124(4)	3807(5)	1971(3)	87(4)
C(50')	6221(4)	3550(4)	1686(3)	76(3)
C(51')	7588(4)	4996(6)	88(2)	43(2)
C(52')	7641(5)	4969(8)	-254(2)	62(3)
C(53')	6962(3)	6466(4)	2035(2)	45(2)

C(54')	6880(3)	6846(5)	2312(2)	65(3)
Rh(1C)	7220(2)	5398(3)	830(1)	27(1)
Rh(2C)	6951(2)	5916(2)	1304(1)	30(1)
Cl(1C)	8715(10)	6704(17)	-427(6)	43(1)
Cl(2C)	7542(7)	9084(9)	1947(6)	42(1)
Cl(3C)	6276(10)	5400(12)	-756(4)	68(1)
Cl(4C)	4819(7)	7832(10)	1475(5)	83(1)
N(1C)	7513(9)	6255(7)	698(4)	25(1)
N(2C)	7220(8)	6777(6)	1144(4)	33(1)
N(3C)	6616(5)	5658(12)	611(3)	29(1)
N(4C)	6350(5)	6125(10)	1084(4)	34(1)
N(5C)	7746(6)	5242(11)	1154(4)	32(1)
N(6C)	7622(5)	5577(11)	1403(4)	37(1)
N(7C)	6864(9)	4693(7)	1066(4)	32(1)
N(8C)	6738(9)	4953(6)	1335(4)	36(1)
N(9C)	7475(9)	4971(12)	385(4)	36(1)
N(10C)	6728(8)	6369(11)	1746(4)	39(1)
C(1C)	7456(17)	6773(9)	877(7)	31(2)
C(2C)	6293(9)	5970(20)	780(5)	32(1)
C(3C)	7795(10)	6346(13)	422(5)	27(1)
C(4C)	8239(16)	6080(40)	404(11)	35(2)
C(5C)	8529(15)	6210(40)	149(12)	35(1)
C(6C)	8360(20)	6590(60)	-96(16)	34(1)
C(7C)	7920(40)	6850(110)	-80(30)	35(2)
C(8C)	7650(30)	6760(80)	180(20)	32(2)
C(9C)	7289(9)	7346(9)	1333(6)	31(1)
C(10C)	6939(11)	7796(16)	1376(10)	37(2)
C(11C)	6994(10)	8301(16)	1591(10)	38(2)
C(12C)	7402(17)	8360(20)	1752(18)	35(2)
C(13C)	7754(12)	7902(16)	1719(11)	37(2)
C(14C)	7700(10)	7400(18)	1504(11)	35(1)
C(15C)	6512(10)	5593(13)	284(4)	31(1)
C(16C)	6480(30)	6151(13)	96(5)	36(2)
C(17C)	6480(30)	6084(15)	-231(5)	45(2)
C(18C)	6400(20)	5474(15)	-356(5)	45(2)
C(19C)	6270(30)	4964(19)	-159(6)	44(2)

C(20C)	6380(30)	4999(15)	155(6)	38(2)
C(21C)	6001(9)	6555(13)	1196(5)	40(2)
C(22C)	5865(14)	7061(19)	1002(9)	49(2)
C(23C)	5498(16)	7460(20)	1091(10)	61(2)
C(24C)	5234(13)	7266(17)	1348(8)	58(2)
C(25C)	5378(15)	6800(20)	1553(9)	55(2)
C(26C)	5749(15)	6400(20)	1459(9)	49(2)
C(27C)	8156(11)	4870(20)	1159(7)	36(1)
C(28C)	8284(14)	4500(20)	889(8)	44(2)
C(29C)	8672(13)	4120(20)	893(9)	48(2)
C(30C)	8968(14)	4100(20)	1153(9)	51(2)
C(31C)	8888(10)	4510(20)	1396(9)	46(2)
C(32C)	8458(11)	4860(20)	1420(7)	40(1)
C(33C)	8350(10)	5270(20)	1675(7)	40(1)
C(34C)	7904(9)	5589(19)	1674(5)	37(1)
C(35C)	7787(11)	6000(20)	1919(7)	46(2)
C(36C)	8091(12)	6070(20)	2173(7)	51(2)
C(37C)	8530(14)	5780(30)	2173(10)	54(2)
C(38C)	8632(12)	5330(20)	1951(7)	50(2)
C(39C)	6732(18)	4061(11)	988(7)	36(2)
C(40C)	6890(20)	3799(17)	704(9)	42(2)
C(41C)	6720(20)	3196(18)	613(9)	49(2)
C(42C)	6510(30)	2800(20)	836(12)	60(2)
C(43C)	6440(30)	3006(13)	1141(11)	58(2)
C(44C)	6530(20)	3660(12)	1216(9)	48(2)
C(45C)	6313(18)	3970(13)	1482(8)	48(2)
C(46C)	6491(19)	4603(12)	1558(7)	39(2)
C(47C)	6390(30)	4890(20)	1848(9)	53(3)
C(48C)	6090(30)	4580(30)	2054(11)	74(3)
C(49C)	5880(30)	3990(30)	1971(13)	87(4)
C(50C)	6030(30)	3670(20)	1709(13)	76(3)
C(51C)	7630(30)	4910(40)	142(9)	43(2)
C(52C)	7740(40)	4860(70)	-192(9)	62(3)
C(53C)	6675(19)	6690(20)	1958(9)	45(2)
C(54C)	6560(30)	7150(30)	2201(13)	65(3)
F(1)	6096(2)	5843(2)	2269(1)	67(1)

F(2)	6056(2)	6202(2)	2766(1)	63(1)
F(3)	5443(2)	6347(3)	2443(1)	93(2)
F(4)	5614(2)	5337(3)	2611(1)	89(2)
B(1)	5803(3)	5936(5)	2516(2)	55(2)
F(5)	8665(1)	6236(2)	1106(1)	51(1)
F(6)	9260(1)	6003(2)	1442(1)	46(1)
F(7)	9249(1)	6961(2)	1189(1)	57(1)
F(8)	8716(1)	6758(2)	1572(1)	59(1)
B(2)	8970(2)	6491(4)	1327(2)	41(2)
F(9)	6836(2)	7736(2)	-128(1)	68(1)
F(10)	6852(2)	7857(2)	401(1)	60(1)
F(11)	7368(3)	8381(4)	86(2)	58(2)
F(11B)	6986(5)	8684(6)	73(3)	70(3)
F(12)	6644(4)	8772(5)	104(3)	80(3)
F(12B)	6282(3)	8197(5)	105(2)	62(2)
B(3)	6889(7)	8184(13)	121(6)	55(2)
B(3B)	6766(9)	8105(15)	116(7)	55(2)
B(4)	8240(5)	7433(7)	2705(4)	67(3)
F(13)	8013(4)	7107(4)	2467(2)	127(4)
F(14)	8640(3)	7692(4)	2609(3)	128(4)
F(15)	7968(2)	7940(3)	2826(2)	82(2)
F(16)	8297(2)	6957(3)	2929(2)	71(2)
F(13B)	8440(11)	7368(17)	2367(6)	70(5)
F(14B)	8741(10)	7639(19)	2818(8)	68(5)
F(15B)	8034(12)	8054(17)	2670(9)	73(5)
F(16B)	8094(13)	7044(18)	2807(9)	74(5)
B(4B)	8326(12)	7514(17)	2671(9)	67(3)

Table S11. Crystal Data and Structure Refinement for **6**.

Report date	2025-12-09	
Identification code	p-CF3-Form	
Empirical formula	C58 H41.40 B2 Cl2 F20 N9 O0.20 Rh2	
Molecular formula	C56 H37.40 F12 N9 O0.20 Rh2, C2 H4 Cl2, 2(B F4)	
Formula weight	1545.93	
Temperature	100.00 K	
Wavelength	0.71073 Å	
Crystal system	Triclinic	
Space group	P-1	
Unit cell dimensions	a = 11.9276(6) Å	α= 95.696(2)°.
	b = 13.9211(8) Å	β= 104.226(2)°.
	c = 19.8745(10) Å	γ = 108.449(2)°.
Volume	2977.7(3) Å^3	
Z	2	
Density (calculated)	1.724 Mg/m^3	
Absorption coefficient	0.756 mm^{-1}	
F(000)	1536	
Crystal size	0.04 x 0.029 x 0.021 mm^3	
Crystal color, habit	red block	
Theta range for data collection	1.981 to 25.422°.	
Index ranges	-14<=h<=14, -16<=k<=16, -23<=l<=23	
Reflections collected	105614	
Independent reflections	10963 [R(int) = 0.1083, R(sigma) = 0.0643]	
Completeness to theta = 25.000°	100.0 %	
Absorption correction	Semi-empirical from equivalents	
Max. and min. transmission	0.0916 and 0.0696	
Refinement method	Full-matrix least-squares on F^2	
Data / restraints / parameters	10963 / 427 / 1028	
Goodness-of-fit on F^2	1.029	
Final R indices [I>2sigma(I)]	R1 = 0.0519, wR2 = 0.1161	
R indices (all data)	R1 = 0.0988, wR2 = 0.1379	
Extinction coefficient	n/a	
Largest diff. peak and hole	1.035 and -0.709 e.Å^{-3}	

Table S12. Atomic Coordinates (x 10^4) and Equivalent Isotropic Displacement Parameters (Å^2 x 10^3) for **6**, where U(eq) is Defined as One Third of the Trace of the Orthogonalized U^{ij} Tensor.

	x	y	z	U(eq)
Rh(1)	5185(1)	8678(1)	7501(1)	30(1)
Rh(2)	5543(1)	7087(1)	7372(1)	31(1)
F(1)	1850(5)	11319(5)	9162(4)	62(2)
F(2)	919(5)	9956(5)	9509(3)	62(2)
F(3)	2585(9)	11070(9)	10213(5)	61(2)
C(3)	4060(20)	8930(20)	8713(5)	36(3)
C(4)	3799(9)	8800(7)	9351(5)	55(2)
C(5)	3171(8)	9351(7)	9616(5)	56(2)
C(6)	2771(12)	10046(11)	9250(5)	46(1)
C(7)	3118(9)	10241(8)	8659(5)	59(2)
C(8)	3768(9)	9687(7)	8400(5)	56(2)
C(9)	2048(9)	10597(8)	9531(5)	54(1)
F(1B)	1440(20)	10980(20)	9177(15)	64(4)
F(2B)	1110(20)	9810(20)	9786(15)	60(4)
F(3B)	2750(40)	11180(40)	10214(18)	56(4)
C(3B)	3950(90)	8890(100)	8659(17)	40(9)
C(4B)	4260(30)	9180(30)	9389(16)	55(2)
C(5B)	3600(40)	9650(30)	9708(15)	56(2)
C(6B)	2740(40)	9990(40)	9283(14)	46(1)
C(7B)	2320(30)	9590(30)	8578(14)	50(7)
C(8B)	2970(30)	9090(30)	8265(14)	45(6)
C(9B)	2030(30)	10500(20)	9616(14)	54(1)
F(4)	4267(5)	2887(4)	9669(3)	91(2)
F(5)	6213(5)	3439(4)	9953(3)	82(1)
F(6)	5180(6)	2560(4)	8931(3)	111(2)
F(7)	8871(7)	13984(5)	9818(3)	78(2)
F(7B)	9500(30)	13930(20)	9922(15)	73(4)
F(8)	8919(7)	14289(5)	8806(4)	76(2)
F(8B)	8300(30)	14190(20)	8888(16)	77(5)
F(9)	10446(6)	14018(5)	9451(4)	92(2)
F(9B)	10170(30)	14110(20)	9091(15)	76(4)

F(10)	11733(4)	6149(3)	8858(2)	64(1)
F(11)	12022(3)	6627(3)	7907(2)	57(1)
F(12)	10700(4)	5151(3)	7857(2)	68(1)
O(1)	4842(16)	10320(14)	7631(10)	30(1)
N(1)	4639(4)	8374(4)	8383(2)	37(1)
N(2)	5191(4)	6902(4)	8314(3)	36(1)
N(3)	6967(4)	9403(4)	8125(2)	33(1)
N(4)	7373(4)	7880(4)	7889(2)	35(1)
N(5)	3539(4)	7614(3)	6884(3)	34(1)
N(6)	3676(4)	6722(4)	6899(2)	33(1)
N(7)	5758(4)	8627(3)	6622(2)	32(1)
N(8)	5848(4)	7717(4)	6498(2)	33(1)
N(9)	5845(4)	5622(4)	7234(2)	32(1)
C(1)	4746(5)	7534(5)	8605(3)	37(1)
C(2)	7724(6)	8862(5)	8169(3)	37(1)
C(10)	5188(5)	6003(5)	8607(3)	37(1)
C(11)	4096(6)	5224(5)	8546(4)	49(2)
C(12)	4089(7)	4345(6)	8815(4)	57(2)
C(13)	5215(7)	4259(5)	9156(3)	47(2)
C(14)	6297(7)	5030(6)	9214(4)	64(2)
C(15)	6289(6)	5901(6)	8949(4)	54(2)
C(16)	5219(8)	3300(6)	9428(4)	64(2)
C(17)	7512(5)	10462(5)	8430(3)	38(1)
C(18)	6875(8)	11106(7)	8267(5)	57(2)
C(18B)	7100(30)	10960(30)	8830(20)	55(8)
C(19)	7413(9)	12164(7)	8526(5)	64(2)
C(19B)	7730(40)	12000(30)	9140(20)	60(8)
C(20)	8593(7)	12573(5)	8959(4)	53(1)
C(21)	9172(8)	11931(6)	9215(5)	56(2)
C(21B)	9110(40)	12070(30)	8490(20)	61(9)
C(22)	8644(8)	10880(6)	8959(5)	53(2)
C(22B)	8490(30)	11080(20)	8181(19)	52(8)
C(23)	9185(8)	13707(6)	9246(4)	66(2)
C(24)	8336(5)	7453(5)	7976(3)	36(1)
C(25)	9110(5)	7541(5)	8641(3)	43(2)
C(26)	10040(6)	7117(5)	8721(4)	48(2)

C(27)	10166(5)	6598(5)	8131(3)	40(1)
C(28)	9407(6)	6521(5)	7467(3)	45(2)
C(29)	8505(5)	6967(5)	7391(3)	43(2)
C(30)	11150(6)	6129(6)	8190(4)	49(2)
C(31)	2444(5)	7690(5)	6513(3)	37(1)
C(32)	2414(5)	8674(5)	6457(3)	43(2)
C(33)	1340(6)	8774(5)	6084(4)	52(2)
C(34)	285(6)	7885(6)	5762(4)	56(2)
C(35)	311(6)	6933(5)	5803(3)	46(2)
C(36)	1399(5)	6791(5)	6181(3)	40(1)
C(37)	1507(5)	5821(5)	6261(3)	38(1)
C(38)	2685(5)	5808(5)	6623(3)	37(1)
C(39)	2865(6)	4878(5)	6741(3)	42(2)
C(40)	1861(6)	3978(5)	6509(4)	49(2)
C(41)	702(6)	3981(5)	6148(4)	49(2)
C(42)	526(6)	4877(5)	6024(3)	45(2)
C(43)	5999(5)	9334(4)	6193(3)	35(1)
C(44)	5886(6)	10286(5)	6366(3)	47(2)
C(45)	6175(7)	11026(5)	5967(4)	58(2)
C(46)	6602(7)	10814(5)	5383(4)	58(2)
C(47)	6687(6)	9882(5)	5198(4)	50(2)
C(48)	6392(5)	9099(5)	5600(3)	38(1)
C(49)	6443(5)	8105(5)	5447(3)	38(1)
C(50)	6131(5)	7405(4)	5903(3)	33(1)
C(51)	6111(5)	6393(5)	5762(3)	40(1)
C(52)	6428(6)	6076(5)	5182(4)	53(2)
C(53)	6743(6)	6768(6)	4734(4)	54(2)
C(54)	6752(6)	7736(5)	4854(3)	45(2)
C(55)	6142(6)	4967(5)	7238(3)	47(2)
C(56)	6588(8)	4095(6)	7241(4)	66(2)
Cl(1S)	9268(2)	9493(2)	6938(1)	90(1)
Cl(2B)	9973(6)	10968(7)	5819(4)	132(3)
Cl(2S)	10287(6)	12786(4)	7639(3)	125(2)
C(1S)	9450(20)	10827(12)	6909(12)	128(5)
C(1SB)	10470(20)	10696(15)	7194(14)	128(5)
C(2S)	10080(20)	11464(12)	7614(11)	121(5)

C(2SB)	9830(30)	11260(20)	6698(10)	121(5)
F(13)	3512(4)	7173(3)	5353(2)	66(1)
F(14)	2247(4)	5526(3)	4906(2)	63(1)
F(15)	2139(4)	6770(4)	4267(3)	87(2)
F(16)	3781(4)	6282(4)	4432(2)	79(1)
B(1)	2910(6)	6433(6)	4719(4)	40(2)
F(17)	4700(10)	11692(8)	7388(5)	88(3)
F(18)	2820(11)	11397(11)	7480(6)	111(3)
F(19)	3763(13)	12790(8)	7050(7)	86(3)
F(20)	3039(13)	11108(6)	6444(4)	94(3)
B(2)	3579(16)	11789(12)	7069(8)	76(2)
F(17B)	4033(16)	11241(11)	6945(11)	116(4)
F(18B)	2959(13)	11961(10)	7575(7)	70(3)
F(19B)	3950(20)	12791(11)	6851(9)	72(3)
F(20B)	2208(13)	11373(10)	6419(6)	87(3)
B(2B)	3302(19)	11828(15)	6965(10)	76(2)

Reference

(S1) Whittemore, T. J.; Xue, C.; Huang, J.; Gallucci, J. C.; Turro, C. Single-Chromophore Single-Molecule Photocatalyst for the Production of Dihydrogen Using Low-Energy Light. *Nat. Chem.* **2020**, *12*, 180–185.
